# Exosomal fragment enclosed polyamine-salt nano-complex for co-delivery of docetaxel and mir-34a exhibits higher cytotoxicity and apoptosis in breast cancer cells

**DOI:** 10.1038/s41598-024-72226-0

**Published:** 2024-09-17

**Authors:** Moumita Basak, Mrunal Kulkarni, Saibhargav Narisepalli, Deepak Chitkara, Anupama Mittal

**Affiliations:** Department of Pharmacy, Birla Institute of Technology and Science (BITS PILANI), Pilani Campus, Pilani, Rajasthan 333031 India

**Keywords:** PAN particles, Exosomal layering, miR-34a, Docetaxel, Co-delivery nanocarrier, Anticancer, Disease prevention, Therapeutics, Cancer

## Abstract

A novel core–shell nanocarrier system has been designed for co-delivery of a small anticancer drug, docetaxel (DTX) and tumor suppressor (TS) miR-34a named as Exo(PAN_34a+DTX_). The core is formed by pH dependent polyamine salt aggregates (PSA) containing both the payloads and the shell is formed by RAW 264.7 cell derived exosomal fragments. Herein, phosphate driven polyallylamine hydrochloride (PAH, MW:17,500 Da) PSA was formed in presence of miR-34a and DTX to form PAN_34a+DTX_. The formulation exhibited pH dependent DTX release with only 33.55 ± 2.12% DTX release at pH 7.2 and 75.21 ± 1.8% DTX release till 144 h at pH 5.5. At 1.21 molar ratio of phosphate to the amine (known as R value), efficient complexation of miR-34a (3.6 μM) in the PAN particles was obtained. PAN_34a+DTX_ demonstrated particle size (163.86 ± 12.89 nm) and zeta-potential value of 17.53 ± 5.10 mV which upon exosomal fragment layering changed to − 7.23 ± 2.75 mV which is similar to the zeta-potential of the exosomal fragments, i.e., − 8.40 ± 1.79 mV. The final formulation Exo(PAN_34a+DTX_), loaded with 40 ng/mL DTX and 50 nM miR-34a exhibited 48.20 ± 4.59% cytotoxicity in triple negative breast cancer (TNBC) cells, 4T1. Co-localization of CM-DiI (red fluorescence) stained exosomal fragments and FAM-siRNA (green fluorescence) in the cytoplasm of 4T1 cells after 6 h of Exo(PAN_FAM_) treatment confirmed the efficiency of the designed system to co-deliver two actives. Exo(PAN_34a+DTX_) also reduced BCL-2 expression (target gene for miR-34a) by 8.98 folds in comparison to free DTX confirming promising co-delivery and apoptosis inducing effect of Exo(PAN_34a+DTX_) in 4T1.

## Introduction

Conventional cancer monotherapies elicit non-selective cytotoxicity accompanied by their immunosuppressive and myelosuppressive activity which leads to adverse effects, secondary cancer induction, metastasis, chemo resistance and cancer recurrence^1^. These inevitable drawbacks could be managed by combination therapy wherein, different pathways could be targeted simultaneously at a significantly lesser dose than recommended for monotherapy regimen of individual drug^2^. Currently, combination therapy with chemotherapeutics and RNA interference (RNAi) has been widely projected as a potential alternative to monotherapy and combination therapy of the chemotherapeutics^3–5^. Among the RNAi, microRNAs (miRNAs) are classified as the non-coding RNAs responsible for regulation of gene expression by post transcriptional modifications. The primary miRNA duplex (pri-miRNA) is transcribed from DNA sequences in the nucleus and converted to the pre-miRNA which is exported to cytoplasm to regulate the respective mRNA translation^6^. As, miRNAs regulate multiple pathways in cancer development and progression, these prove beneficial in maintaining the sensitivity of tumor towards chemotherapeutics either by restoring tumor suppressor activity (by TS miRNA mimic) or suppression of oncogenic dominance (by miRNA inhibitor)^6–8^. MRX 34a, the first miRNA mimic to reach phase I clinical trial in 2013, clearly proved that, even at a suboptimal dose, miRNA can turn on the cellular threshold of tumor suppression^9^. Interestingly, miR-34a is a well reported TS miRNA, which is found suppressed in various types of cancer including both solid tumors and blood cancers. miR-34a is reported to induce apoptosis, cell cycle arrest and growth inhibition by downregulating the target genes including cyclin dependent kinase (CDK4/6), silent information regulator 1 (SIRT1), BCL-2, MYC and MET, and is considered as the transcriptional target of p53^10,11^. Recently, miR-34a has also proven to sensitize the resistant breast cancer cells towards DTX giving impetus to the field of combination delivery of miRNA and  a chemotherapeutic^12^.

Exogenous miRNA delivery faces critical hurdles, including poor cellular uptake (due to negative charge), off target effects, short half-life and limited stability in blood stream^13–16^. Encapsulation of miRNAs in nanoparticles by nanoprecipitation or emulsion techniques results in low encapsulation efficiency, attributed to the quick diffusion of miRNA into the water phase because of its high affinity towards water, although nanoparticles improve the tissue distribution and site-specific localization of miRNA^17^. The importance of efficient delivery of DTX and miR-34a in breast cancer is exemplified by some literature reports. A folate conjugated cationic lipo-polymer has been reported to form NC with miRNA at N/P ratio of 8:1 with 94.8% DTX payload. This system showed improved cytotoxicity (~ 5 folds), and apoptosis (~ 2.0 folds) when compared to the free DTX in breast cancer cells^4^. Another novel core–shell nanoformulation was reported for the co delivery of miR-34a and DTX in breast cancer wherein, 83.46 ± 2.36% DTX was encapsulated in the lipidic core and 13.91 ± 0.39% miR-34a was electrostatically complexed in the cationic bovine serum albumin (BSA) shell of the nanoformulation. The resultant system delivered the payload in the cytoplasm as indicated by appearance of a yellow fluorescence emerging from the overlay of Cyanine 5-RNA (red fluorescence) and Coumarin 6 (C6, green fluorescence) as the model replacements of the miRNA and DTX in 4T1 cells. Further, the in vivo efficacy study confirmed ~ 1.74 fold higher tumor volume reduction by combination therapy in comparison to the free DTX in 4T1 tumor bearing animals^18^. In an interesting study carried out to explore the application of exosomes for the RNAi delivery, cationic BSA (cBSA) was synthesized to prepare the cBSA/siRNA nanoplexes which were further serially extruded with exosomes (redispersed in hypotonic buffer) 100 times to entrap the cBSA/siRNA nanoplexes within the exosomal layer. In a time-dependent biodistribution study, cBSA/siRNA@Exo showed 2.9 times improved uptake in lung tumor in comparison to the cBSA/siRNA@liposomes^19^. Exosomes, being biogenic nanocarriers with distinct paracrine signaling property, have demonstrated enormous potential in designing of the novel drug delivery systems. RAW 264.7 cell derived exosomes depicted improved pharmacokinetic profile of DTX when encapsulated into the exosomes (Exo-DTX) in comparison to the marketed formulation Taxotere^20^. They have self-resealable property with anionic surface charge which proves beneficial in masking the cytotoxicity of cationic nanoformulations as widely reported^21,22^. Yet, the application of these exosomal membranes in the delivery of small molecules and miRNAs is still in infancy.

In this context, “Tandem self-assembly” is considered as one of the relatively less explored **core–shell** delivery systems in which cationic polyamines form a nanosized **core** structure (polyamine-salt aggregates or PSA) in the presence of the multivalent anionic salts, and the **shell** material (either nanoparticles, polymer, or biomolecules) of reverse ionic charge form the capsule surrounding the core by means of electrostatic interaction^23^. Apart from electrostatic interaction, hydrophobic interaction and hydrogen bonding are also observed playing an important role in PSA formation^24^. Interestingly, a wide variety of cargo molecules have already been delivered using PSA including, small dye molecules, drugs, contrast agents, siRNA, enzymes and proteins^25–28^. In 2010, Wong et al. reported a PSA system to encapsulate the imaging molecule, indocyanin green (ICG) in the Polyallylamine (PAH)-phosphate spherical aggregate, which was further coated with anti-EGFR (epidermal growth factor receptor) antibodies to selectively target the EGFR overexpressing malignant tumor in three carcinoma cell lines, 1483 (human head and neck squamous cell), SiHa (human cervical squamous cell), and 435 (cancerous human breast cell) depicting variable expression of EGFR^26^. Almost a decade later, Andreozzi et al*.* reported application of PSA for siRNA delivery wherein, they developed a pH responsive polyamine phosphate nanocarrier (PAN) by leveraging the electrostatic interaction between amine groups of PAH (pK_a_: 8.8) and the phosphate group of the optimized phosphate buffer (PB) to complex the negatively charged siRNA. They also proved that PAN remained stable at physiological pH but dissembled only when pH of dispersant media attained a value below pH 6 or above pH 9. Although they successfully delivered the green fluorescence protein (GFP) tagged siRNA through PAN in the GFP-A549 cells; the cellular viability was found compromised within 16–72 h, when 6.6 × 10^–5^ mM monomers were used for PAN formation, possibly, due to high positive charge of the nanoparticles^28,29^. This system has never been explored for delivery of DTX. DTX possesses one hydroxyl group at C-10 and forms hydrogen bond with tertiary amine group of Dipalmitoylphosphatidylcholine (DPPC)^30^ and thus offers a high possibility of hydrophobic interaction between the primary amine of Polyallyamine and DTX but it has not been explored so far.

Considering these facts, we aimed to explore the phosphate driven PSA to co-load DTX and miR-34a in the core designated as PAN_34a+DTX_. Phosphate buffer saline (PBS) acts as a source of phosphate ions required to crosslink Poly(allylamine) hydrochloride (PAH, pKa = 8.8); its composition was optimized for co-loading DTX and miR-34a in the noncomplex. RAW 264.7 cell derived exosomes were utilized to prepare exosomal fragments to form the shell of the core, PAN_34a+DTX_ and yield the final formulation Exo(PAN_34a+DTX_). It was hypothesized that the presence of exosomal layer would improve the cellular uptake of the PAN_34a+DTX_ along with protecting the cells from the toxicity resulting from the cationic noncomplex. Additionally, the “proton sponge” effect of the polyamines (due to their characteristic cationic nature) in the acidic environment of endosomes would help the formulation to burst open and release the payload in cytoplasm^31^. The main aim of this work was to explore and optimize this novel strategy to co-deliver DTX and miR-34a, characterize the formulation and evaluate its efficacy in Triple negative breast cancer (TNBC) 4T1 cells.

## Materials and methods

### Materials

Polyallylamine hydrochloride (PAH, MW: 1.75 × 10^4^ g/mol), sodium phosphate dibasic (Na_2_HPO_4_), potassium phosphate monobasic (KH_2_PO_4_), hydrochloric acid (HCl), sodium hydroxide (NaOH), sodium chloride (NaCl), and DEPC (diethylpyrocarbonate, molecular grade) were obtained from CDH (New Delhi, INDIA). Polyelectrolyte stock solutions and all the subsequent diluted precursor solutions were prepared in Milli-Q deionized water (dI). Docetaxel Trihydrate (DTX) was kindly donated by Fresenius Kabi (New Delhi, India). Formulations were prepared in the DEPC containing dI water. FAM-siRNA was obtained from GeneCust, Europe, while, hsa-miRNA-34a-5p mimic, mirVana miRNA mimic negative control, LIPOFECTAMINE-2000 and CellTracker CM-DiI, PIERCE BCA protein assay kit and 4% paraformaldehyde (#ALF-J61899-AK) were purchased from ThermoFisher Scientific (USA). For SDS-PAGE and Western blot purpose, we procured N,N,N’,N’-Tetramethylethylenediamine (TEMED) from Thermo Scientific (Waltham, USA), Tween 20 (#TC287) from Himedia laboratories (Maharashtra, India) and rest of the reagents, i.e., Ammonium Persulphate (#1610700EDU), Clarity Western ECL Substrate (#1705060), PRECISION PLUS PROTEIN standards (#161-0394), and IMMUNO-BLOT PVDF membrane were procured from Bio-Rad (California, USA). While, RIPA lysis buffer (TCL 131), calcium chloride dihydrate (GRM 399) and Tris base (MB311) were procured from Himedia (Maharashtra, INDIA), Protease inhibitor cocktail powder (#SRE 0055-1BO) was bought from Sigma-Aldrich (St. Louis, MO, USA). The primary antibodies Alix mAb (#92880), TSG101 mAb (#72312S) and secondary antibodies, anti-rabbit IgG HRP linked antibody (#7074P2), anti-mouse IgG HRP linked antibody (#7076 s), and β-actin (#3700) were procured from Cell Signalling Technology (Danvers, USA) and mouse CD63 (sc-365604) mAb was obtained from Santa Cruz Biotechnologies (Texas, USA). For agarose gel electrophoresis, Agarose (RM6249), RNA loading dye (R1386-5VL) and TBE buffer 10X (T4415) were purchased from Sigma Aldrich (USA). Ethidium bromide (RM 813) and Heparin sodium salt (TC138) were procured from Himedia (INDIA) and Ribonuclear low range RNA ladder (SM1831) was procured from Thermo Fischer (USA). For qRT-PCR, GAPDH and BCL-2 primers (sequences are mentioned in Table 1) were procured from Integrated DNA Technologies (Lowa, USA), GeneSure first strand cDNA synthesis kit was purchased from Puregene (New Delhi, INDIA), and iTAQ Universal SYBR green Supermix was from Bio-Rad (California, USA). Additionally, Rat IFNγ (900-M109) and TNFα (900-M73) Mini ABTS ELISA Development kit for the ELISA assay were procured from Pepro Tech (NJ, USA).

**Table 1:** Sequences of primers used for qRT-PCR.

Gene	Forward sequence	Reverse sequence
BCL-2	5′-CCTGTGGATGACTGAGTACC-3′	5′-GAGACAGCCAGGAGAAATCA-3′
GAPDH	5′-TGCATCCTGCACCACCAACT-3′	5′-AGCCTGCTTCACCACCTTC-3′

### Cell culture

Murine TNBC 4T1 cells and RAW 264.7 cells were procured from Regional Centre of Biotechnology (Haryana, India) and National center for cell science, NCCS (Maharashtra, India) respectively. We are grateful to Professor Avinash Bajaj for providing the 4T1 cells. Exosome depleted FBS was prepared *in-house* by ultracentrifugation of a mixture of FBS and PBS (FBS:PBS = 3:7) at 1,20,000 $\times$ g and 4 °C for 18 h. Dulbecco’s modified Eagle’s medium (Gibco DMEM, high glucose) and Fetal bovine serum (FBS) were purchased from GIBCO (Invitrogen Inc. Gibco BRL, USA). Both DMEM and minimum essential medium (MEM) incomplete media, Penicillin plus streptomycin solution, 4′,6-diamidino-2-phenylindole (DAPI), and 3-(4,5-dimethylthiazol-2-yl)-2,5-diphenyltetrazolium bromide (MTT) were bought from Sigma-Aldrich (St. Louis, MO, USA).

### Methods

#### Optimization and development of formulations

**Fig. 1:**
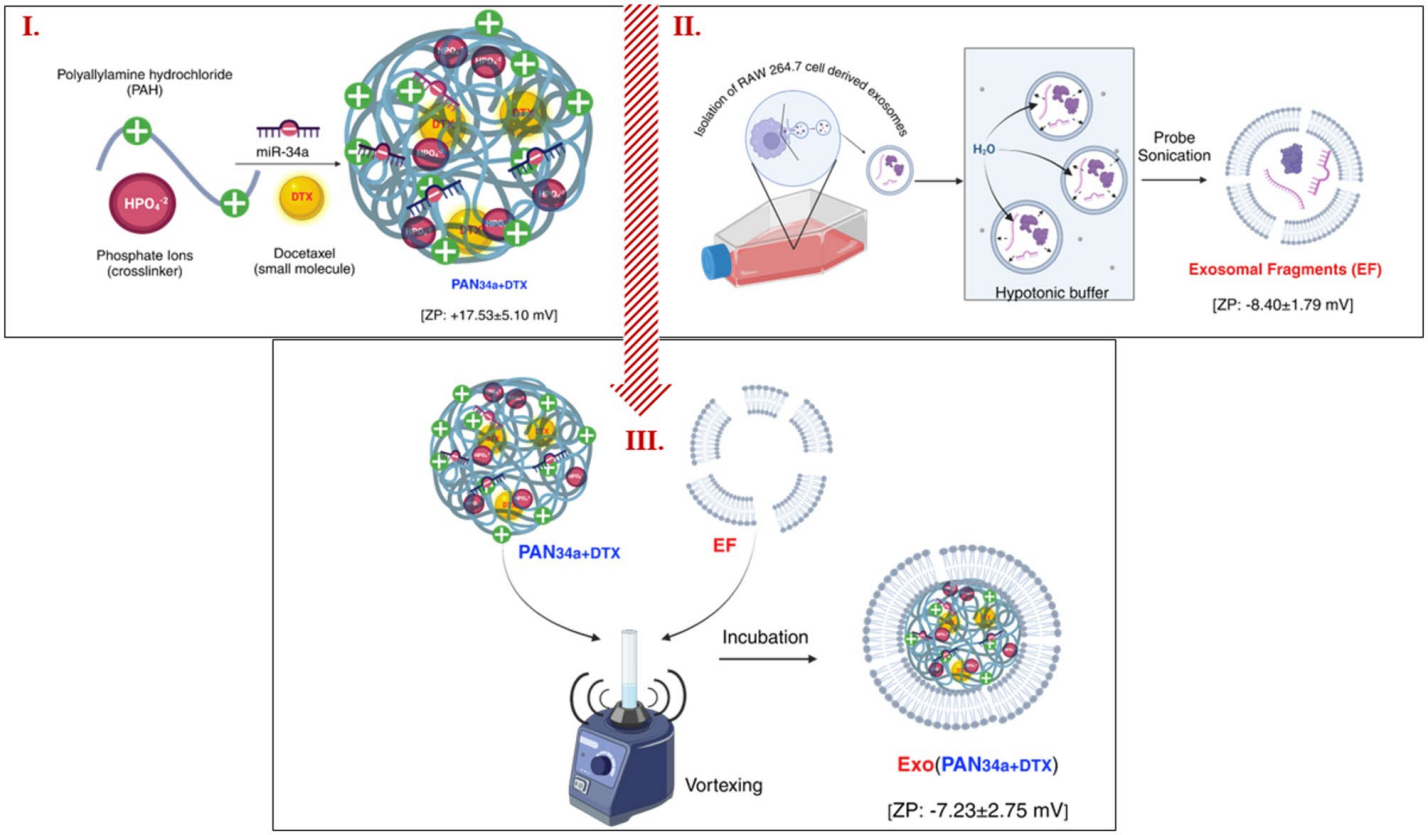
Fabrication of Exo(PAN_34a+DTX_). Step I: Preparation of PAN_34+DTX_, step II: Preparation of exosomal fragments (EF) and, step III: preparation of Exo (PAN_34a+DTX_).

Fabrication of Exo(PAN_34a+DTX_) was carried out in three major steps, involving the preparation of (I) *PAN*_*34a*+*DTX*_ (II) exosomal fragments (EF) followed by, (III) Exo(PAN_34a+DTX_) (Fig. 1**)**.

##### Development and optimization of PAN_34a+DTX_

PAN_34+DTX_ was prepared by adding miR-34a (~ 0.5 μg) and DTX (400 ng from 12 μg/mL aqueous solution) together into PBS (containing 5 mM Na_2_HPO_4_, 3 mM KH_2_PO_4_ and 10 mM NaCl). Next, 3 μl of PAH (10 mg/mL solution) was added to the reaction mixture to achieve an effective concentration of 1 mg/mL PAH. Appearance of spontaneous cloudiness indicated the formation of PAN_34a+DTX_ formulation. The reaction mixture was allowed to stand at room temperature for 10 min for aging and then centrifuged at 5000 rpm for 5 min to remove the particles with higher particle size. The supernatant with small sized particles was further processed for exosomal layering.

In this process, the composition of PBS (molar ratio of Na_2_HPO_4_: KH_2_PO_4_ and amount of NaCl), PAH concentration (1–0.01 mg/ml) and effect of dilution on the properties of PAN were optimized to achieve an appropriate R value (ratio of total negative charges from the multivalent salt to the total positive charge of the polyamine) as indicated in Eq. (1). Two important factors i.e., the composition of PBS and PAH concentration were screened as detailed in Table 2, which was further followed by understanding the pH dependency of PAN_DTX_ formation. For the optimization of PAN_RNA_ formulation, total RNA isolated from RAW 264.7 cells was quantified and utilized as a replacement of miR-34a. Further, the complexation of miR-34a in PAN_34a_ was confirmed by agarose gel electrophoresis. PAN_34+DTX_ used for all further experiments was prepared with the optimized process parameters and material attributes.

**Table 2:** Representative batches of the blank PAN to optimize the R ratio.

S. no.	PAH as polyamine (mg/mL)	Phosphate ion (cross-linker)		Ratio of Na_2_HPO_4_ and KH_2_PO_4_	R ratio
		[Na_2_HPO_4_] (mM)	[KH_2_PO_4_] (mM)		
B1	1.0	5	1	5:1	0.93
B2	1.0	5	2	5:2	1.12
B3	1.0	5	3	5:3	1.214
B4	0.1	5	3	5:3	12.14
B5	0.01	5	3	5:3	121.4

1 $$\text{R}=\frac{[Phosphate ]\times {n}^{-}}{[PAH]\times {n}^{+}}$$

##### Isolation and preparation of RAW 264.7 cell derived Exosomal Fragments (EF)

*Isolation of the RAW 264.7 cell derived exosomes* RAW 264.7 cell derived exosomes were isolated by differential centrifugation of 200 mL of RAW 264.7 conditioned media (CM) collected from 175 cm^2^ flask. The CM was processed step-wise 500 $\times$ g, 2000 $\times$ g and 13,000 $\times$ g for 10 min, 15 min and 30 min respectively at 4 °C. At each step, the supernatant was collected and finally subjected to ultracentrifugation at 120,000 $\times$ g at 4 °C for 2 h. Once the exosomes were pelleted down, supernatant was discarded and the exosomes were washed with an excess of PBS (10 mM) at 120,000 $\times$ g and 4 °C for 2 h. The final pellet was redispersed in 400 µl of PBS. The uniformly redispersed exosomes were then processed for EF preparation.

*Preparation of EF* For the preparation of EF, RAW 264.7 cell derived exosomes were resuspended in ice-cold hypotonic solution, i.e. Tris-calcium buffer (TC buffer; 0.01 M Tris and 0.001 M calcium chloride, pH 7.4), supplemented with EDTA free protease inhibitor cocktail and stored at 4 °C overnight. This treatment emptied the payload of exosomes and yielded the ghost exosomal membrane (Exo_TC_)^19^. The Exo_TC_ were further settled down by ultracentrifugation at 1,80,000 × g, 4 °C for 3 h and redispersed in the RNAse free water before proceeding for the EF preparation. The Exo_TC_ were further probe sonicated (Vibra-Cell, Sonics, USA) under cold conditions at 30% amplitude, 10 s on/off cycle twice for disrupting the exosomal integrity without completely rupturing them. As an alternative to probe sonication, sequential extrusion though 200 nm and 100 nm pore size for 10 cycles was also carried out for EF preparation. The details of the batches prepared are mentioned in Table 3.

**Table 3:** Comparative particle size, absolute intensity, PDI and zeta-potential of exosomes by dynamic light scattering (DLS).

Batch	Specification	Particle Size (nm)	Absolute intensity (Kcounts/s)	PDI	Zeta-potential (mV)
	Naïve Exosomes	208.7 ± 36.19	NA	0.256 ± 0.03	− 10.268 ± 3.66
B6	Exosomes (TC buffer)	257.83 ± 51.06	354,769.6 ± 82,618	0.24 ± 0.023	− 20.6 ± 0.87
B7	Exosome ghost (Exo_TC_)	189.92 ± 8.47	352,927 ± 11,772.5	0.23 ± 0.01	− 11.7 ± 0.57
B8	Exo_TC_ → Sonication	156.29 ± 0.07	339,743 ± 2853.02	0.20 ± 0.02	− 11.3 ± 0.29
B9	Exo_TC_ → Extrusion (200 nm)	201.26 ± 1.00^**@**^	144,673.2 ± 34,226.53	0.26 ± 0.06	− 12.9 ± 1.30
B10	Exo_TC_ → Extrusion (200 nm → 100 nm)	207.3 ± 44.05^**$**^	204,523 ± 37,135.5	0.29 ± 0.1	− 13.27 ± 0.47

^@^Particle size with average 79.7% peak intensity.

^$^Particle size with average 53.5% peak intensity.

##### Preparation of Exo(PAN_34a+DTX_) and Exo(PAN_FAM+DTX_)

Exo(PAN_34a+DTX_) was prepared by vortexing freshly prepared EF from ~ 12 μg equivalent Exo_TC_ with PAN_34a+DTX_ (containing 0.5 μg miR-34a and 400 ng of DTX) for 5 min at RT and the volume of the formulation was made upto 100 μL with RNAse free water. The exosomal layering was confirmed by reduced haziness of the final formulation and was allowed to stand at 4 °C for 1 h to allow efficient layering of the particles by EF. The protocol as discussed for the preparation of Exo(PAN_34a+DTX_) was followed for the preparation of Exo(PAN_FAM+DTX_) formulation only by replacing miR-34a with 0.5 μg FAM-siRNA.

#### Characterization of the formulations

All the formulations were characterized for particle size (nm), absolute intensity (Kcounts/s) and zeta-potential (mV).

##### Particle size distribution, absolute intensity, and zeta-potential

All the formulations were characterized by DLS using Anton Paar Litesizer 500 using backscattering mode (173° scattering angle), with 15 s equilibration time, 10 runs for each analysis with 10 s for each run at 25 °C. The absolute intensity indicated the light scattering capacity of the particles which is directly proportional to the number of particles. Absolute intensity (Kcounts/s) is generated by the instrument depending upon the sample concentration. Further, zeta-potential measurements were performed in total 50 runs with 10 s for each run at 25 °C.

##### Characterization of EF by western blot

Exosomes (~ 100 µg intact exosomes) were redispersed in 50 µl of RIPA buffer supplemented with protease inhibitor cocktail and probe-sonicated to release the exosomal proteins as exosome lysate (EL). RAW 264.7 cells, maintained in DMEM supplemented exo-free FBS were washed thrice in PBS and then lysed to prepare the cell lysates i.e., CL_F_ by sonication (10 s on/off, 2 cycles, 30% amplitude) at 15 °C. CL_F_, EL and EF (~ 30 µg equivalent protein) were resolved in 12.5% and 15% SDS PAGE, and transferred in polyvinylidene fluoride membrane. After blocking the blots in 3% BSA in TBST solution, the presence of exosomal marker proteins, CD63, Alix and TSG 101 and housekeeping protein β-actin was ascertained by probing the blots in CD63 (1:500), Alix (1:2000), TSG 101 (1: 2000) and β-actin (1:1000) mice monoclonal antibodies overnight followed by 3 h incubation with secondary HRP linked anti-mouse/rabbit IgG antibody (1:1000). Thereafter, the blots were developed using Clarity Western ECL Substrate (#1705060) in ChemiDocXRS + (Bio-Rad, California, USA).

##### Morphology by field emission scanning electron microscopy (FESEM)

The morphology of the naive exosomes, Exo_TC_, EF, PAN_34a+DTX,_ and Exo(PAN_34a+DTX_) was ascertained by FESEM. For the naive exosomes and Exo_TC_, samples (~ 50 µg protein) were redispersed in 1.3% paraformaldehyde (PFA) solution and spread onto the coverslip. For EF (~ 50 μg protein), sample was smeared on the coverslip as soon as Exo_TC_ was probe sonicated. Likewise, FESEM sample for PAN_34a+DTX_ was prepared one-day prior to analysis by putting a drop of the formulation on a clean coverslip and air drying overnight. FESEM sample of Exo(PAN_34a+DTX_) was also processed similar to EF. The sample smears were washed with dI water thrice and air-dried overnight at RT. Next day, all samples were gold-sputtered (2–5 nm) and analyzed using 20 kV beam in FESEM. Alongside the morphology, the individual particles from each sample (n = 50) were also analyzed for size and expressed as mean ± SD for the characterization of particle size.

##### Functional characterization of PAN_DTX_

* In-vitro stability study* In this seven-day long stability study of PAN_DTX_, 5 mL formulation was prepared in triplicate and stored at 4 °C and stability samples were withdrawn at pre-determined time-points, i.e., 0.5, 1, 4, 7, 12, 24, 48, 72, 96, and 144 h. The samples were analyzed for the particle size, zeta-potential and %EE using DLS and HPLC to check the stability of the PAN_DTX_ formulation^32^.

* In-vitro release study* The release pattern of DTX from the PAN_DTX_ formulation was determined using dialysis bag method. In this method, free DTX and PAN_DTX_ were dispersed in dI water and transferred to a regenerated cellulose dialysis tube bag (SNAKESKIN dialysis tubing, Thermo Fisher, MWCO3500 Da). The dialysis bag was immersed in 5 mL of release media (PBS + 0.1% Tween 80) at two different pH values (7.2 and 5.5), and at each time-point, 2 mL of release media was withdrawn and replaced with fresh media. The amount of DTX released from PAN_DTX_ at each time-point in both the release media was analyzed using a validated HPLC/UV method^32^.

* In-vitro hemocompatibility study* PAN_DTX_ was tested for its hemocompatibility as PAH is a synthetic and cationic polymer. For this assay, blood was collected from the Wistar rats in a collection tube containing 10% w/v EDTA solution as the anti-coagulant and centrifuged at 2500 rpm at RT for 5 min. The RBC pellets were washed with excess PBS till the supernatant became colorless. Next, the RBC were redispersed in PBS (250 μL) and incubated with different samples, including PBS (negative control), 0.1% Triton-X-100 (positive control), Free DTX (~ 100 ng/mL), blank PAN particles and PAN_DTX_ (100 ng/mL DTX) for 30 min. Thereafter, the samples were centrifuged to pellet down the RBCs and the supernatants were analyzed using Epoch ELISA plate reader at 540 nm. The % hemolysis was calculated using Eq. (2).

2 $$\text{Hemolysis }(\text{\%}):\frac{{OD}_{sample}-{OD}_{negative \,control}}{{OD}_{positive \,control}-{OD}_{negative \,control}}\times 100$$

##### Characterization of miR-34a complexation by gel retardation assay

The miR-34a complexation capacity of the PAN_34a+DTX_ and Exo(PAN_34a+DTX_) has been evaluated by gel retardation assay wherein, the miR-34a content (0.05–0.5 μg) and PAH concentration (0.01–1 mg/mL) were varied. Additionally, the complete complexation of miR-34a was confirmed by performing the heparin competition assay. Herein, different PAN formulations containing 0.2 μg miR-34a and 0.01–1 mg/mL PAH, were prepared and incubated with heparin (2 I.U.) for 1 h at RT. Formulations (in the presence and absence of heparin) were electrophoresed simultaneously in 2% agarose gel containing 0.5 μg/mL ethidium bromide (EtBr). The electrophoresis was carried out in 0.5 × Tris- Borate-EDTA buffer at 100 V for 20 min. The run was further followed by visualization on ChemiDocXRS + .

#### In vitro efficacy studies

TNBC 4T1 cells were cultured in DMEM (high glucose), supplemented with 10% FBS, and penicillin–streptomycin (100 IU/mL) in complete culture media in an incubator at 37 °C in a humidified atmosphere with 5% CO_2_. Cells were kept for 12 h to adhere post seeding in each study before performing the experiment.

##### Transfection and uptake efficiency

4T1 cells (1.5 × 10^4^ cells/well) were seeded over a coverslip in a 6-well plate and allowed to attach and form a monolayer. The culture media was replaced with Opti-MEM media 1 h prior to the treatment and the cells were further incubated with FAM-siRNA Lipofectamine 2000 (positive control), PAN_FAM_, Exo(PAN_FAM_), and Exo(PAN_FAM+DTX_) for 6 h. In the final formulation, PAN_FAM+DTX_ was prepared containing 40 ng/mL DTX and 50 nM FAM-siRNA.

CM-DiI (red fluorescence) stained EF were prepared by incubating exosomes (~ 100 μg protein) with CM-DiI for 1 h at 4 °C and ultracentrifuged at 180,000 × g for 2 h to pellet down CM-DiI stained exosomes. These CM-DiI stained exosomes were further redispersed in the hypotonic solution and processed for preparing the CM-DiI stained EFs.

Post treatment, the cells were washed with PBS thrice, fixed with 2% PFA for 15 min at RT followed by staining with DAPI. The samples were further washed with PBS and analyzed by confocal microscopy.

##### In-vitro cytotoxicity study

2.5 × 10^3^ cells were seeded in 96-well plate and allowed to adhere for 12 h before being treated with different formulations. The cells were treated with formulations i.e., blank PAN, free DTX, free miR-34a, EF, PAN_DTX_, PAN_34a_, Exo(PAN_DTX_), Exo(PAN_34a_), and Exo(PAN_34a+DTX_). All the formulations were loaded with 40 ng/mL DTX and 50 nM miR-34a. The cells were treated for 48 h and then were subjected to MTT assay.

##### Inflammatory cytokine release

Briefly, 4T1 cells (2 × 10^6^ cells/well) were seeded in 6-well cell culture plate and allowed to adhere overnight and different treatments, i.e., free DTX, blank PAN, EF (~ 6 μg and ~ 12 μg protein), PAN_DTX_, PAN_34a_, PAN_34a+DTX_, Exo(PAN_DTX_), Exo(PAN_34a_), and Exo(PAN_34a+DTX_) containing 40 ng/mL DTX and 50 nM miR-34a were given for 48 h. At the end of 48 h, the CM from cells was collected and centrifuged to expel out the dead cells and cellular debris at 3500 rpm, 4 °C for 5 min. The supernatants were collected and ELISA assay of tumor necrosis factor (TNF-α) and interferon (IFN-γ) was carried out.

##### Gene expression analysis

Following the same experimental protocol as mentioned in the previous section, the cells were trypsinized and washed thrice with sterile PBS and dispersed in 1 mL of RNA-XPRESS Reagent (Himedia) and stored at − 20 °C overnight. After isolating the total RNA, concentration of RNA in each sample was quantified by Nanodrop. The cDNA synthesis was carried out using GENESURE First Strand cDNA Synthesis Kit following manufacturer’s protocol and quantified with Nanodrop. 200 ng equivalent cDNA was utilized to determine the cycle threshold (C_T_) values using Real-time PCR for target gene BCL-2 and housekeeping gene GAPDH. The C_T_ value of the BCL-2 for each sample was normalized with respect to the C_T_ value of the GAPDH. Gene quantification was performed using Universal SYBR green Supermix. The fold change of the BCL-2 expression was expressed by calculating 2^−∆∆C^_T_ values.

### Statistical analysis

The data has been represented as mean ± standard deviation as processed in GraphPad Prism (Version 5.0, USA). The difference between two groups was compared using Student’s *t-*test, comparison between multiple groups was carried out using one-way ANOVA followed by a Tukey’s test. Value of *p* < 0.05 was considered as statistically significant.

## Results

### Development and optimization of *PAN* formulations

#### Determination of the suitable R ratio for blank *PAN* formation

**Fig. 2:**
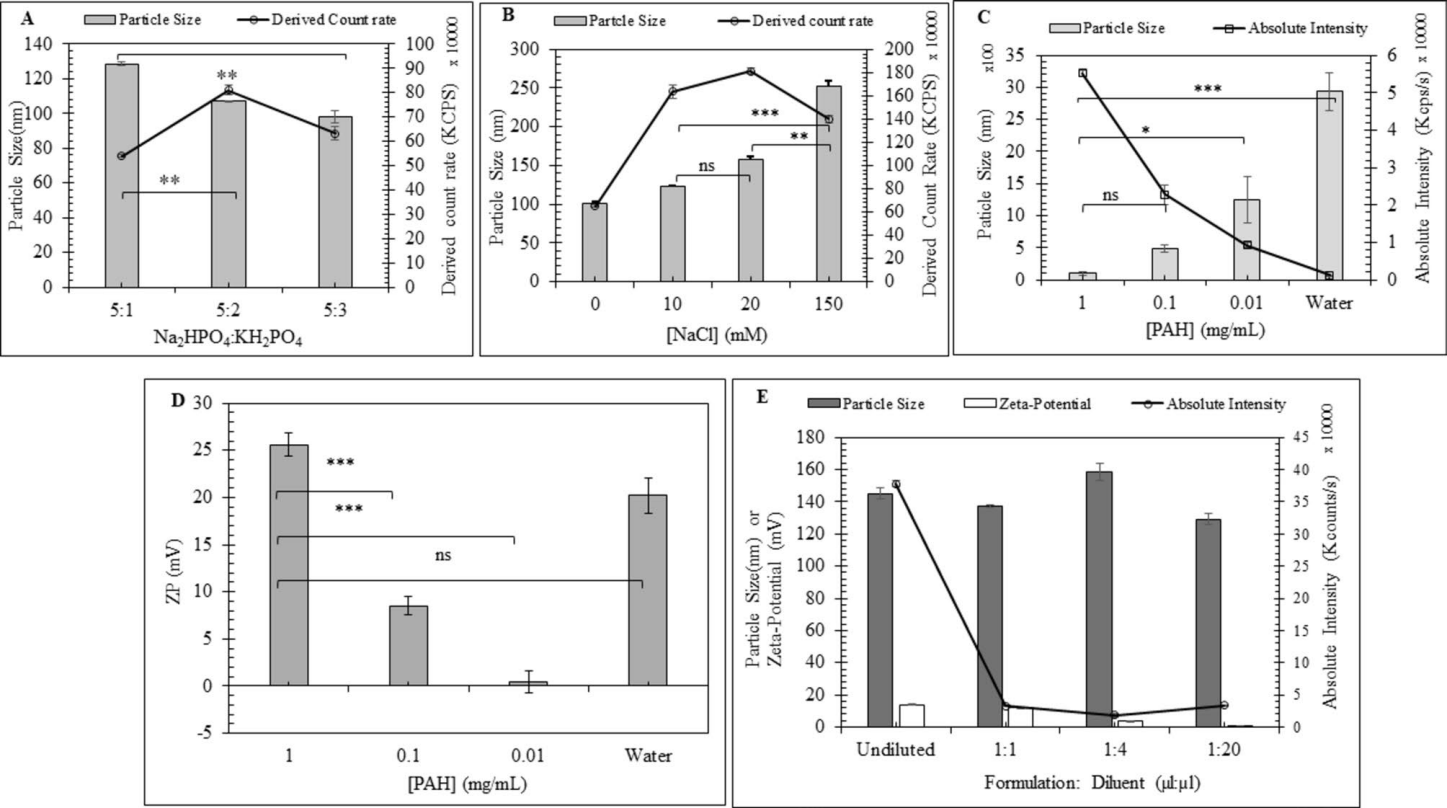
Optimization of blank PAN based on particle size, zeta-potential and absolute intensity (Kcounts/s). (**A**) Effect of molar ratio of Na_2_HPO_4_:KH_2_PO_4_ (varied from 5:1 to 5:3) at 10 mM sodium chloride. (**B**) Effect of concentration of sodium chloride (0/ 10/ 20/ 150 mM) with optimized Na_2_HPO_4_:KH_2_PO_4_ = 5:3 (**C**) Effect of PAH concentration (0.01–1 mg/mL) prepared in optimized PBS (Na_2_HPO_4_:KH_2_PO_4_: NaCl: 5:3:10). The PAN particles thus formed were compared to PAH (1 mg/mL) in water. (**D**) Effect of PAH concentration on the zeta-potential of the blank PAN when the concentration was varied between 0.01-1 mg/mL in modified PBS and compared with PAH (1 mg/mL) in dI water (**E**) Effect of dilution. All experiments were performed in n = 3 (three independent experiments), statistical significance was ascertained by one-way ANOVA with Tukey’s comparison test, *p < 0.05, **p < 0.01, ***p < 0.001.

It is well reported in literature that PSA formation and its meta-stable condition is regulated by the molecular weight of polyamine, R-ratio, ageing time, storage temperature and dilution^23^. Taking clues from the literature, the buffer composition, polyamine concentration, and dilution were further optimized in this work to obtain the R ratio at which the formed PAN particles would remain stable for a sufficient duration of time to enable further processing following ‘single parameter change at once’ method. The parameters were varied as mentioned in Table 2 and the effect is depicted in Fig. 2.

As given in Table 2, Batches# B1-B3 demonstrated a significant role of phosphate ion as a cross-linker as also seen in Fig. 2A wherein, R≈1.21 resulted in PAN formation in 10 mM NaCl with minimum particle size, i.e., 98.135 ± 3.75 nm and ~ 1.75 folds more derived count rate than the particles formed with same R ratio in absence of NaCl. PAN formed in the presence of NaCl (10–150 mM) compared to the PAN particles formed in absence of NaCl showed gradual enhancement in the derived count rate in a concentration dependent manner (Fig. 2B) wherein, addition of NaCl contributed additional ionic strength to the media thus stabilizing the particles^33^. A slight increase in the size of PAN particles i.e., 122.25 ± 0.96 nm with ~ 2.5 folds’ more derived count rate (KCPS) after the incorporation of 10 mM NaCl in the modified PBS favored the presence of NaCl in PBS giving the optimized PBS composition as Na_2_HPO_4_: KH_2_PO_4:_ NaCl::5:3:10. Further, varying PAH concentration in the range of 1.0–0.01 mg/mL (5.74 × 10^–5^ M–0.057 × 10^–5^ M) resulted in 10 and 100 folds increased R ratio (Table 2, #B3–B5) and was found to have a significant impact on the particle size, absolute intensity and zeta-potential of the blank PAN; herein the optimized composition of PBS has been used as the dispersant media. As indicated in Fig. 2C, with increasing R ratio and decreasing polyanion concentration the particle size drastically increased and absolute intensity decreased. But, the observed intensity of PAN prepared with 0.01 mg/mL PAH in optimized PBS was higher than the absolute intensity observed when the same amount of PAH was added in deionized water. This clearly proved that the formation of PAN particles required the presence of phosphate ions as a cross-linker for PAH wherein, the molar ratio of the phosphate ions and amines (PAH) not only determine the tendency to form aggregates spontaneously but also the stability and physical characteristics of the PAN particles. Interestingly, PAN showed increase in zeta-potential value from 0.44 ± 1.16 mV to 25.6 ± 1.27 mV when PAH concentration was increased from 0.01 and 1.0 mg/mL in optimized PBS at same R ratio. (Fig. 2D) After the formation of blank PAN, effect of dilution was evaluated as indicated in Fig. 2E. It was observed that dilution of blank PAN particles by increasing the volume of the diluent (dI water) resulted in same particle size with drastically decreased absolute intensity as expected. Interestingly, particles diluted in 1:1 v/v ratio were found equivalent in size to the undiluted PAN particles (except for the decrease in absolute intensity) and hence 1:1 v/v ratio was considered best suited among all the dilution ratios. This indicated that particles remained stable even after dilution and inspite of their metastable nature did not exhibit self-aggregation.

#### PAN_DTX_

**Fig. 3:**
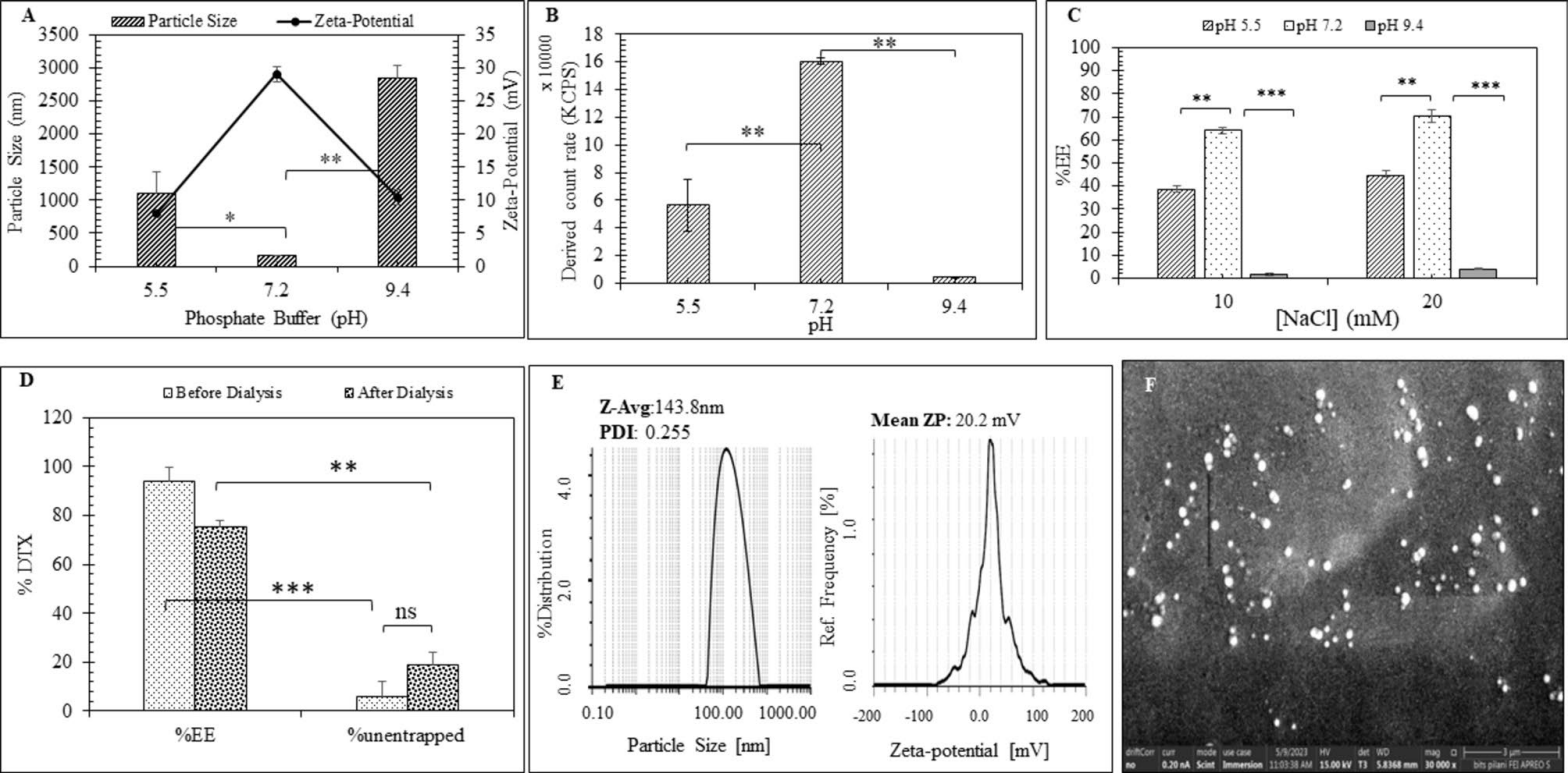
Optimization and characterization of PAN_DTX_ formulation. Effect of pH (5.5, 7.2, and 9.4) of reaction medium on, (**A**) particle size (nm) and zeta-potential (mV) and, (**B**) derived count rate (KCPS). (**C**) Effect of pH and concentration of NaCl on %EE of DTX, (**D**) DTX quantified in the formulations before and after dialysis, (**E**) representative particle size distribution and zeta-potential graphs and, (**F**) Representative morphology of the PAN_DTX_ using FESEM at magnification 30,000X, scale bar 3 μm. Data are represented as mean(n = 3) ± SD, where one-way ANOVA with Tukey’s test was used for determination of the statistical significance; *p < 0.05, **p < 0.01 and ***p < 0.001.

The formation and the %EE of DTX in PAN_DTX_ was found pH dependent (Fig. 3A,B). PAN_DTX_ formed at pH 7.2 showed particle size of 158.3 ± 9.19 nm, zeta-potential of 29.1 ± 1.13 mV (Fig. 2E) with 77.5% EE. It was also observed that at pH 5.5 and 9.4, PAN_DTX_ exhibited very high particle size with significantly lowered zeta-potential values (Fig. 3A). This might be attributed to inefficient PAN_DTX_ formation at these two pH values, as also supported by the significant reduction in the derived count rate (KCPS) at these two pH values (Fig. 3B). The observed pH dependency was further correlated with the %EE of DTX in the presence of different concentrations of NaCl (Fig. 3C). Previously, it was demonstrated that presence of NaCl facilitated the PAN particle formation (Fig. 2B) however, it was also anticipated that increasing NaCl might alter the DTX entrapment efficiency significantly. Experimentally, %EE was found maximum at pH 7.2 and was not significantly affected by NaCl concentration. The minimum %EE was seen at pH 9.4 and this could be attributed to the formation of large sized particles (Fig. 3A), which were later removed during centrifugation at 5000 rpm. The efficient entrapment of DTX in PAN_DTX_ formed at pH 7.2 was proved by dialyzing PAN_DTX_ into 30 mL of water for 1 h to expel out the dissolved but unentrapped DTX. Figure 3D has clearly indicated that DTX unentrapped in the formulation was found to be 5.9 ± 6.0% and ~ 18.79 ± 5.3% before and after dialysis. Further, PAN_DTX_ were found to be spherical but not very compact particles when viewed under FESEM (Fig. 3F). 50 such individual particles gave mean size of 163.3 ± 42.64 nm by FESEM which was in agreement with the size estimated by DLS (Fig. 3E).

#### PAN_RNA_ and PAN_DTX+RNA_

**Fig. 4:**
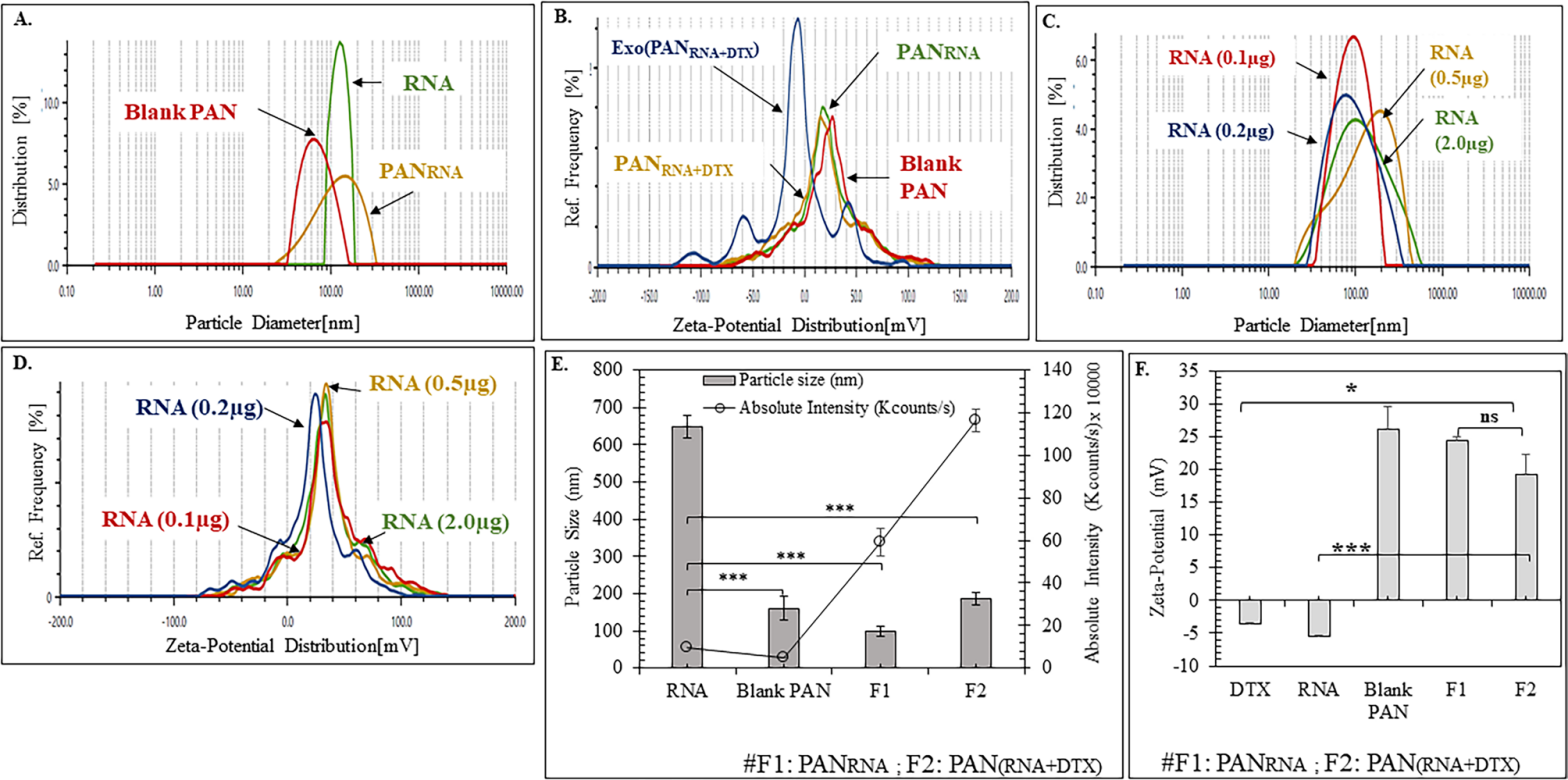
Characterization of PAN_RNA_ formulation by particle size and zeta-potential measurement (**A**) Particle size of blank PAN, RNA (2 μg) and PAN_RNA_ at R ratio of 1.214 in modified PBS. (**B**) Zeta-potential of blank PAN, PAN_RNA_, PAN_RNA+DTX_, and Exo (PAN_RNA+DTX_) indicating efficient formulation development leveraging electrostatic interaction. Effect of varying the content of RNA on (**C**) particle size (nm) and (**D**) zeta-potential (mV) of the PAN_RNA_ at R ratio 1.214 in modified PBS. Representative (**E**) particle size and absolute intensity and, (**F**) zeta-potential (mV) confirming the formation of PAN_RNA_ and PAN_RNA+DTX_*.* All data are represented as mean (n = 3) ± SD, where one-way ANOVA with Tukey’s test was used for the determination of the statistical significance, where *p < 0.05, **p < 0.01 and ***p < 0.001.

The formation of PAN_RNA_ and PAN_RNA+DTX_ was confirmed by an increase in particle size and decrease in zeta-potential values as compared to the blank PAN (Fig. 4A,B). The successful exosomal layering of PAN_DTX+RNA_ was also confirmed by the zeta potential values wherein, Exo(PAN_RNA+DTX_) demonstrated zeta potential of − 11.4 mV, which is the characteristic surface charge of the EFs (Fig. 4B). It was also observed that increasing amount of RNA (0.1–2 μg) resulted in formation of PAN_RNA_ with varied particle size at constant R ratio wherein, the larger particles were formed with the higher amount of RNA (Fig. 4C). Unlike the particle size, the surface potential of PAN_RNA_ was not found dependent on the content of RNA used (Fig. 4D). As indicated in Fig. 4E, PAN_RNA+DTX_ did not exhibit significantly different particle size than the blank PAN but exhibited significantly higher absolute intensity than the blank PAN and PAN_RNA_. Figure 4F proved the complexation of RNA and encapsulation of DTX in the PAN particles as seen by drastic change in the zeta-potential. While, RNA exhibited zeta-potential of − 5.54 ± 0.219 mV, upon complexation to form PAN_RNA_ and PAN_DTX+RNA,_ the zeta-potential values increased to 24.35 ± 0.64 mV and 19.25 ± 3.04 mV; clearly proving the efficient encapsulation of DTX and RNA in the PAN particles (26.05 ± 3.6 mV).

#### PAN_34a+DTX_ and Exo(PAN_34a+DTX_)

**Fig. 5:**
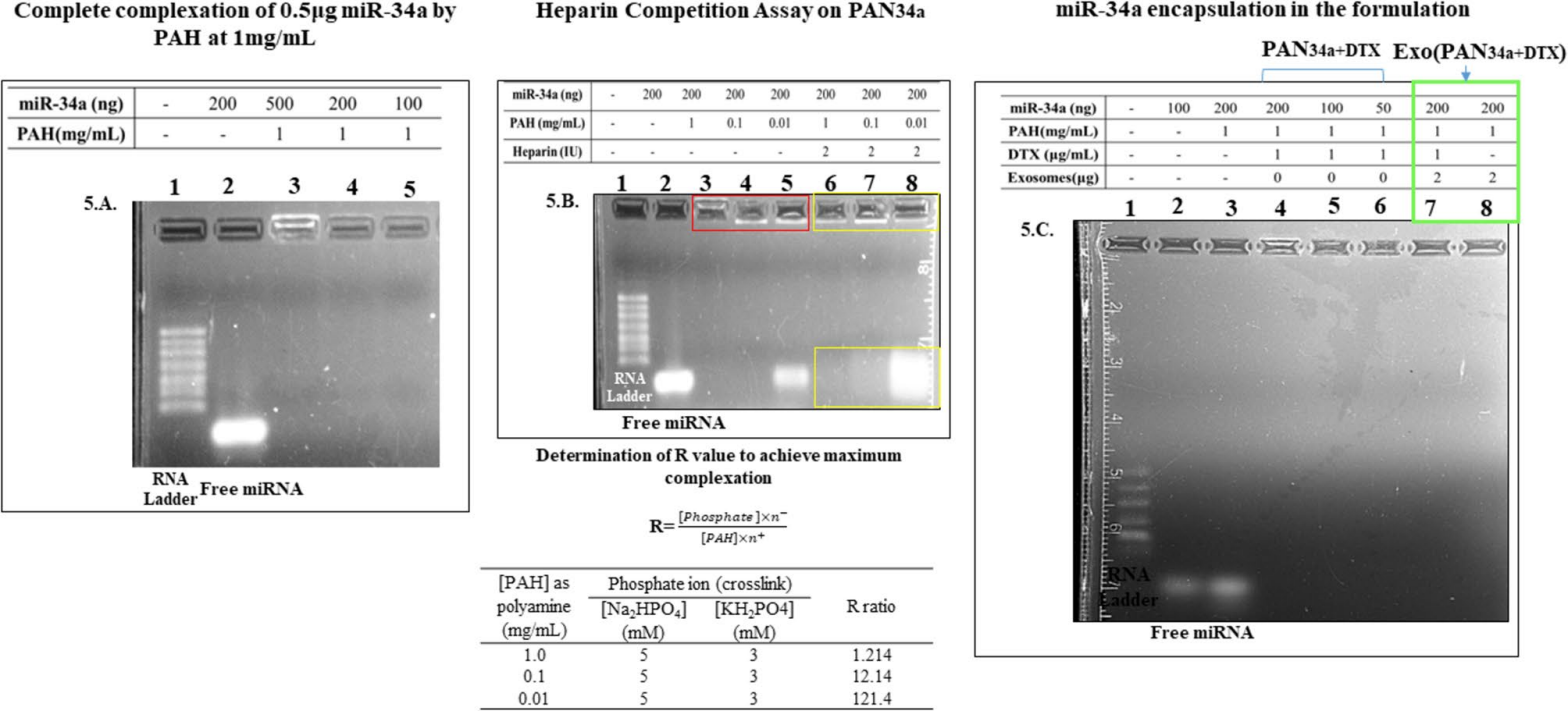
Characterization of PAN_34a+DTX_ and Exo (PAN_34a+DTX_). (**A**) Effect of varying the amount of miR-34a (100–500 ng) on the formation of PAN_34a_ at R≈1.2 (**B**) Effect of the PAH concentration on miRNA complexation in the absence (lanes 3–5) and presence of heparin (lanes 6–8). (**C**). Complexation of miRNA demonstrated by gel retardation assay.

After initial optimization of *PAN*_*RNA*_, RNA was replaced by the miRNA of our interest i.e., miR-34a. Using the gel retardation assay (Fig. 5A), the complexation efficiency of PAN particles (R≈1.2) with 100–500 ng miR-34a in PAN_34a+DTX_ was evaluated. Initially, it was found that the optimized R ratio (followed throughout the formulation development) was able to completely complex even higher amount of miR-34a (500 ng) as visualized in **lane 3** as well. Further, the heparin treatment clearly indicated that the interaction between miR-34a and PAN particles was strong enough to hinder miRNA release in presence of its competitor polyanion heparin (Fig. 5B). Also, it indicated that R ratio (~ 1.214) of the formulation had an important role in the complexation of miR-34a especially as indicated in lanes 3 and 4 vs lane 5. With increasing R ratio (by decreasing the polyanion concentration), the miR-34a complexation was found compromised in **lane 5** (R = 121.4) in comparison to **lane 3** (R = 1.21). The inference was further confirmed by heparin treatment of the same formulation which resulted in the release of highest amount of miR-34a in lane 8.

Finally, the characterization of PAN_3a+DTX_ and Exo(PAN_3a+DTX_) by gel retardation assay is shown in Fig. 5C. The complexation efficiency of PAN particles (R ≈ 1.2) with 50–200 ng miR-34a in PAN_34a+DTX_ and Exo(PAN_34a+DTX_) was evaluated. All the formulations were found retarded in the well and showed no migration of miRNA unlike free miRNA (100–200 ng) in lane 2 and 3. Surprisingly, the fluorescence intensity of retarded miR-34a was only visible in **lane 4** but not in **lane 5–6** which presumably might be attributed to a stronger interaction between miR-34a and the PAN particles (as miRNA amount was decreased from 200 to 50 ng in lanes 4, 5 and 6 respectively) which hindered free access of the miRNA to EtBr mediated intercalation^34^. Further, formulation prepared using higher amount of miRNA (200 ng) was seen clearly retarded in the well (Lane 4) as indicated by the faint fluorescence observed in the well, but was not prominently visible for the formulations prepared with lower amount of miRNA (50 and 100 ng in lane 5 and 6).

Further, miR-34a was replaced with FAM-siRNA to prepare PAN_FAM_ and PAN_FAM+DTX_ which were further used for transfection and uptake assays.

### Characterization of the EF

The impact of the sequential process on the preparation of EF by sonication and extrusion was compared by means of particle size, absolute intensity, zeta-potential and protein estimation of batches# B6-B10, as indicated in Table 3.

**Fig. 6:**
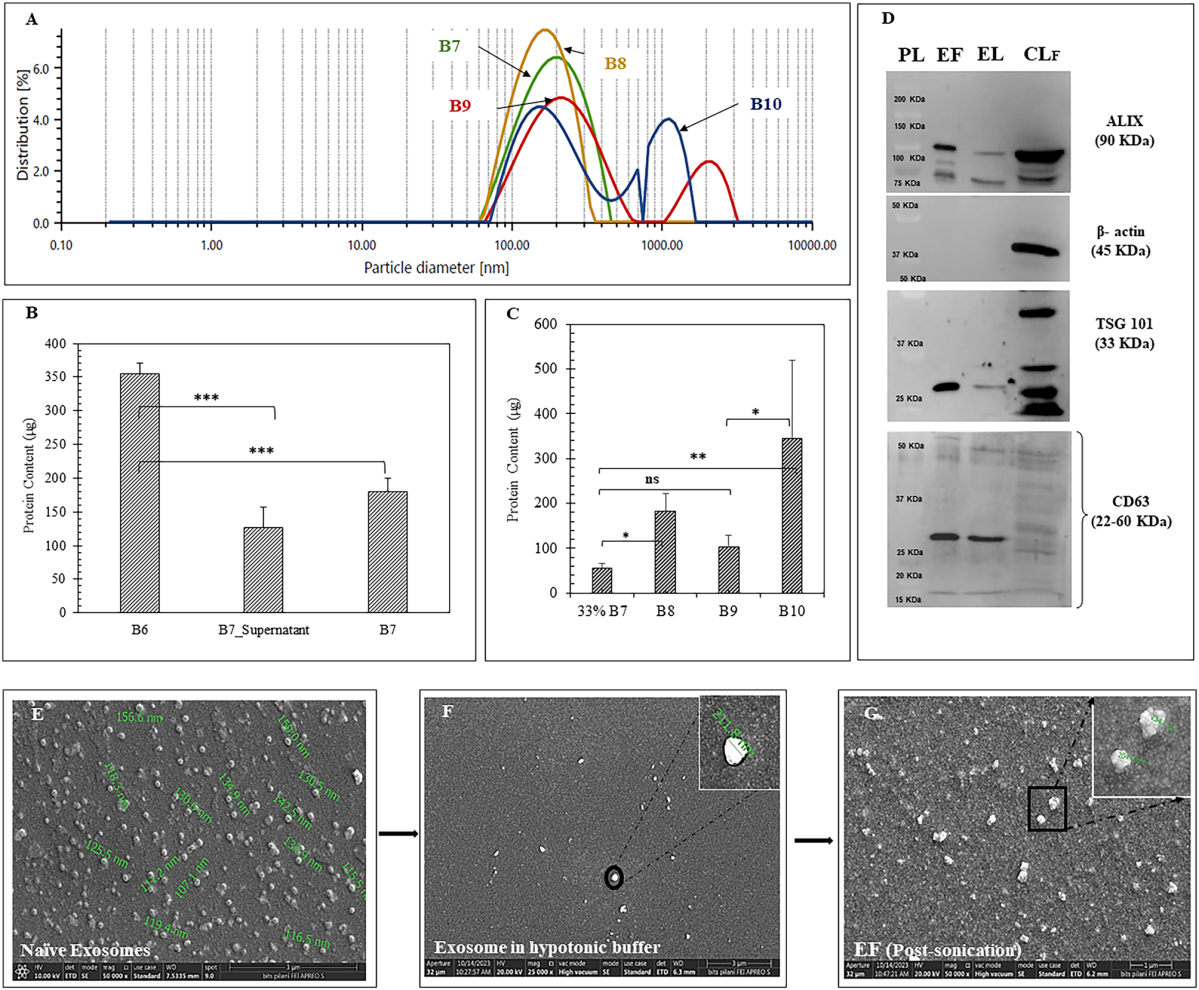
Characterization of EFs. (**A**) Particle size distribution plot demonstrating the effect of two methods, i.e., sonication and sequential extrusion on EF preparation. (**B**) Quantification of protein (μg) in EF preparation (**B7)**, data represented as mean (n = 4) ± SD, (**C**) quantification of protein (μg) in EF preparation by sonication (**B8**) and sequential extrusion through 200 nm and 100 nm (200 nm → 100 nm) pore diameter (**B9–B10**), data represented as mean (n = 4) ± SD. (**D**) Characterization of exosomal protein expression in EF with respect to EL and CL_F_. Characteristic morphology of (**E**) naïve exosomes, (**F**) exosomes in hypotonic Tris/CaCl_2_ buffer (pH 7.4), and (**G**) morphology of exosome fragments prepared by sonication. Statistical significance was ascertained by one-way ANOVA with Tukey’s comparison test, *p < 0.05, **p < 0.01, ***p < 0.001.

The detailed characterization of the naïve exosomes have already been reported in our previous work^20^. For the preparation of EF, Naïve exosomes were kept in hypotonic Tris/CaCl_2_ buffer (TC) overnight to produce B6, which was further ultracentrifuged to pellet down the exosomal vesicles, denoted as **Exo**_**TC**_** (B7)**_._ Later, B7 was processed further by two well-known methods, i.e., sonication (at 30% amplitude) and extrusion (sequentially through 200 nm and 100 nm pore diameter for 10 times) to prepare B8 and B9-B10 respectively. The overall impact of the process could be ascertained from Table 3 and Fig. 6 (A-F). Table 3 clearly concluded that naïve exosomes underwent swelling in B6 from 208.7 ± 36.19 nm to 257.83 ± 51.06 nm upon being treated with hypotonic TC buffer. The movement of solvent into the exosomes towards the osmotic gradient might be responsible for the observed size increase. Figure 6A clearly highlighted that sonication mediated EF preparation did not completely rupture the phospholipidic bilayer, instead disrupted the intactness of the exosomal boundary as indicated in Fig. 6(E–G). Table 3 indicated B6 exhibits maximum negative zeta-potential value of − 20.6 ± 0.87 mV, this could be attributed to the proteins released from the exosomes, as seen in Fig. 6B showing the presence of 127.3 ± 29.18 μg equivalent protein in the supernatant (B7_supernatant). This might be due to the built-in osmotic pressure causing occasional exosomal membrane rupture. Figure 6B indicated segregation of total protein from B6 (exosomes kept in hypotonic buffer overnight) into UC-supernatant and B7 upon ultracentrifugation. This observation proved the osmotic pressure mediated release of proteins from B6. In Fig. 6C, sonication definitely reduced the particle size in B8 but could not alter the absolute intensity or zeta-potential, which proves the exosomal ability to withstand the harsh experimental conditions. Also, extrusion ruptured the B7 to an extent that two distinct size populations were observed, which could be confirmed by the increased PDI and enhanced surface negativity owing to the presence of exosomal proteins. In comparison to B7, both sonication (B8) and extrusion (B9 and B10) resulted in excessive protein release from the exosomes as indicated by Fig. 6C. Further, EF were characterized for the presence of exosomal membrane proteins, TSG101, ALIX and CD63 and absence of β-actin in comparison to the exosome lysate (EL) and RAW 264.7 cell lysate (CL_F_) (Fig. 6D and S1)_._ Interestingly, EL and EF expressed identical proteins wherein, EF showed higher expression of the target proteins than EL and absence of β-actin in EF clearly indicated that the EF originated from the exosomes and not from the cells. Also, the identical pattern of protein expression in EF and EL proved that the process parameters followed did not alter the physiological features of the exosomes. The morphological changes in the exosomes during the sequential process of EF preparation were clearly indicated by the presence of multiple particle size peaks, increased PDI and enhanced zeta-potential negativity in Fig. 6A which could be correlated to the FESEM analysis. This also revealed enhanced particle size of naïve exosomes (Fig. 6E) after being kept overnight in hypotonic buffer upto 257.83 ± 51.06 nm (Fig. 6F). Post-sonication, B8 showed distorted exosomal boundary with characteristic unevenness in individual structure of exosomes, which is visibly different in shape than the Exo_TC_ (Fig. 6G).

### Characterization of Exo(PAN_34a+DTX_)

**Fig. 7:**
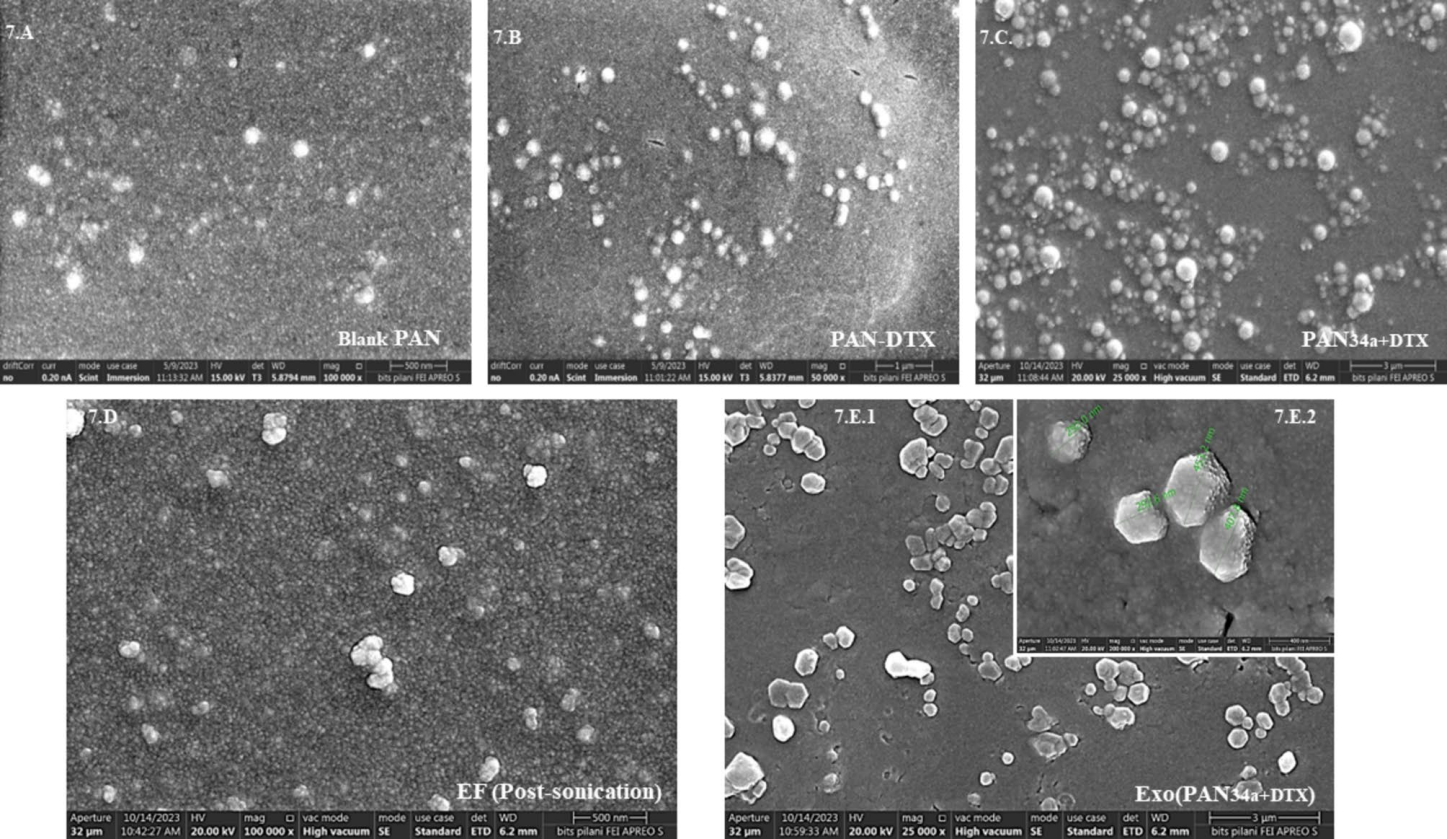
Comparative morphological characterization of the formulations by FESEM. (**A**) Blank PAN, (**B**) PAN_DTX_, (**C**) PAN_34a+DTX_ and (**D**) EF, (**E1**) Exo (PAN_34a+DTX_), scale bar: 3 μm. (**Inset E2**) Single particle analysis was carried out at 200,000 X. Scale bar 400 nm for Exo (PAN_34a+DTX_) inset.

Exo(PAN_34+DTX_) formulations were formed by electrostatic interaction between the positively charged PAN_34a+DTX_ and negatively charged EF. The final formulation was characterized by the particle size, zeta-potential and FESEM for morphology analysis. As indicated in Fig. 5C, miR-34a was seen retarded in well of the **lane 4** but fluorescence could not be observed in lane 7 and 8 containing Exo(PAN_34a+DTX_) and Exo(PAN_34a_), which is only possible if EtBr could not access the miRNA complexed in the final formulation, as the particles were layered with the exosomal fragments. Later on, the final formulation was characterized for the morphological changes before and after exosomal layering that is; PAN_34a+DTX_ and Exo(PAN_34a+DTX_). Morphologically, PAN_34a+DTX_ were spherical shaped but not very compact particles, whereas the EF showed non-uniform particle size and shape, which together resulted in formation of non-uniform but compact Exo(PAN_3a4+DTX_). Table 4 shows that the core/shell strategy resulted in Exo(PAN_34a+DTX_), with zeta potential value of − 7.23 ± 2.75 mV, which is similar to that of EF i.e., − 8.40 ± 1.79 mV, since EF form the shell of the core–shell formulation. In comparison to the positive charge of PAN_34a+DTX_ (17.53 ± 5.10 mV), the negative zeta potential of Exo(PAN_34a+DTX_) indicates the successful layering of EF on PAN_34a+DTX_ formulation. In fact, considering the particle size of different groups (Table 4, and Fig. 7), it could be concluded that, Exo(PAN_34a+DTX_) are non-spherical, and heterogeneous and possibly enclosed several PAN_34a+DTX_ particles resulting in high particle size (393.87 ± 127.89 nm) in comparison to PAN_34a+DTX_ formulation.

**Table 4:** Comparative particle size, PDI and zeta-potential of PAN_34a+DTX_ and Exo (PAN_34a+DTX_).

Formulation	Blank PAN	PAN_34a+DTX_	EF	Exo(PAN_34a+DTX_)
Particle size (nm)	116.83 ± 28.62	163.86 ± 12.89	180.73 ± 127.71	393.87 ± 127.89
Zeta-potential (mV)	24 ± 4.35	17.53 ± 5.10	− 8.40 ± 1.79	− 7.23 ± 2.75

Interestingly, PAN_DTX_ (Fig. 7B) and PAN_34a+DTX_ (Fig. 7C) were formed as spherical particles with relatively higher compactness than the blank PAN particles (Fig. 7A). In comparison to the spherical core, EF (Fig. 7D) demonstrated a non-uniform structure. After the exosomal layering of the core formulation, a characteristic morphological change is clearly evident (Fig. 7E) when compared to both PAN particles and the EF.

### In vitro functional assays

#### Functional studies for PAN_DTX_

**Fig. 8:**
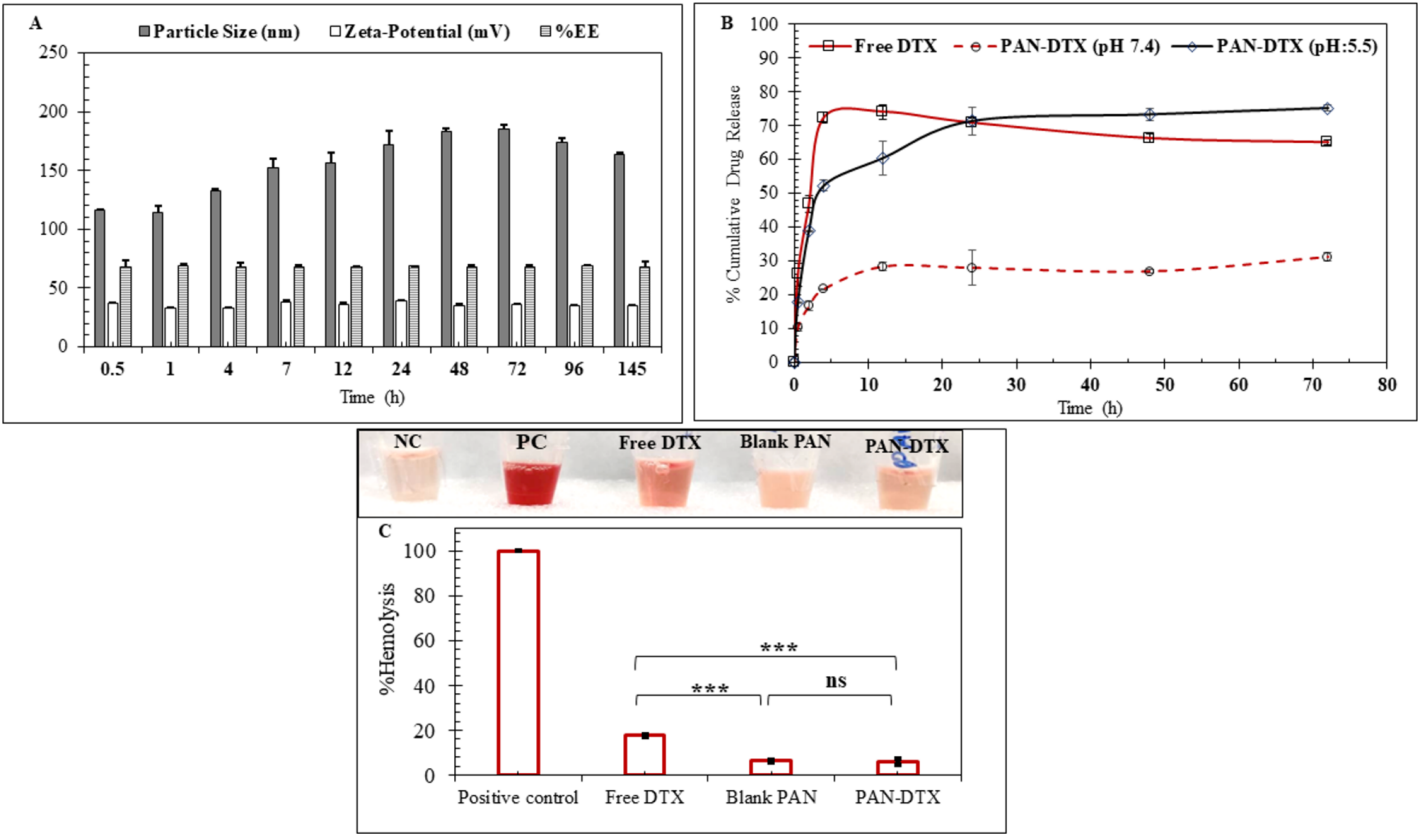
In vitro functional studies of PAN_DTX_ formulation. (**A**) Seven-day long stability study at 4 °C. All data are represented as mean (n = 3) ± SD, (B) in vitro pH dependent release of DTX at pH = 5.5 and 7.4.% Cumulative drug release is expressed as mean $\text{(n=3})\pm$ SD, (**C**) in vitro hemocompatibility study wherein, % haemolysis was evaluated for free DTX (100 ng/mL), Blank PAN, PAN_DTX_ (100 ng/mL) and expressed as mean (n = 3) ± SD, one-way ANOVA with Tukey’s test was used for determination of the statistical significance, where *p < 0.05, **p < 0.01 and ***p < 0.001.

As indicated in Fig. 8A, a seven-day long stability study at 4 °C was performed which indicated slight increase in the particle size of PAN_DTX_ within initial 12 h from 116.5 ± 0.70 nm to 156.5 ± 9.19 nm and then the size remained stable till 144 h. Unlike the particle size, the zeta-potential and the %EE of the PAN_DTX_ remained constant throughout the study.

Figure 8B indicates the most important property of PAN_DTX_, i.e., pH dependent release of DTX from the formulation. It is seen that free DTX showed 26.05 ± 2.63% and 72.41 ± 2.57% release within 30 min and 4 h respectively, thereafter a plateau phase persisted till 24 h followed by an insignificant decrease to 63.31 ± 1.70% at 72 h. In comparison to free DTX, PAN_DTX_ at pH 7.4 showed only 10.35 ± 0.39% and 21.79 ± 1.45% of DTX release within 30 min and 4 h respectively. PAN_DTX_ demonstrated overall constant release till the end of the study with a cumulative drug release of 33.55 ± 2.12% within 72 h. With the change in pH from 7.4 to 5.5, an initial burst release was observed within first 4 h, wherein 17.75 ± 0.44% and 52.33 ± 0.17% DTX was released within 30 min and 4 h respectively and maximum 75.21 ± 1.8% DTX was released within 72 h. This clearly indicated that, PAN_DTX_ would remain intact at physiological pH (pH 7.2) and would only release DTX in endo-lysosomal compartment and acidic tumor microenvironment.

Figure 8C highlights the physiological safety of the formulation upon coming in contact with blood components. This study revealed that, PAN_DTX_ was less hemotoxic than the free DTX wherein, free DTX exhibited 17.69 ± 0.69% and PAN_DTX_ 5.93 ± 1.2% hemolysis in comparison to the positive control (RBCs treated with 0.1% Triton-X-100).

#### Functional studies for Exo(PAN_34a+DTX_)

##### In vitro transfection efficiency and uptake

**Fig. 9:**
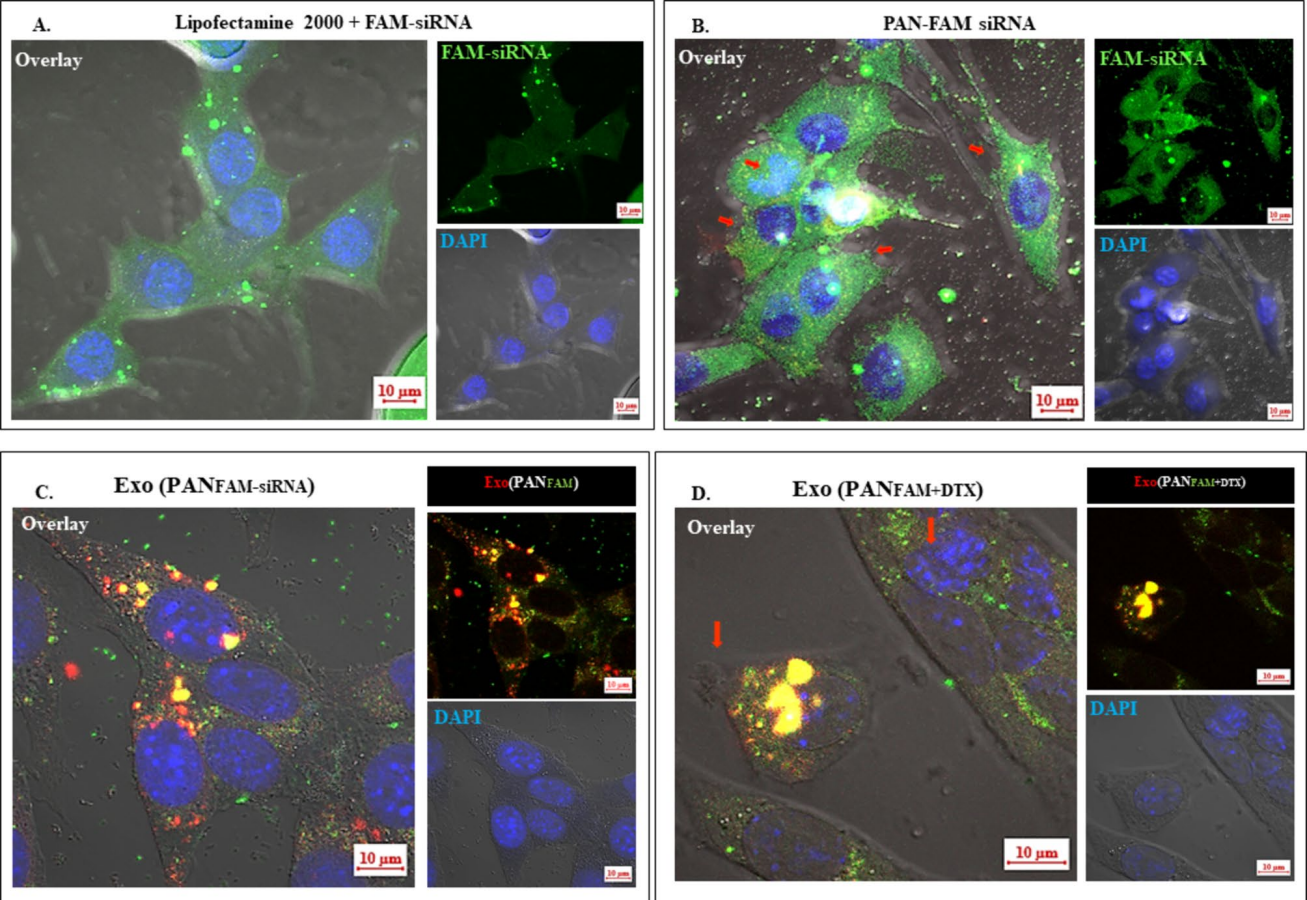
In vitro transfection efficiency and uptake of different formulations in 4T1 cells. FAM siRNA transfection mediated by, (**A**) Lipofectamine 2000 (**A**, as positive control) and, (**B**) PAN particles. (**C)** Representative uptake efficiency of FAM-siRNA by Exo(PAN_FAM_) wherein, CM-DiI stained exosomes were utilized to prepare EF and used for formulation development. The overlay of red (CM-DiI) and green (FAM siRNA) fluorescence produced yellow fluorescence within the cells indicating successful uptake of the formulation by 4T1 cells. (**D**) The CM-DiI stained Exo(PAN_FAM+DTX_) showed both uptake (as indicated by yellow fluorescence) and cytoskeletal deformation (due to DTX) in 4T1 cells. Magnification 630X and scale bar 10 μm.

In vitro transfection efficiency and uptake of FAM-siRNA using commercially available transfecting agent Lipofectamine 2000 was compared qualitatively with the designed PAN particles (Fig. 9A,B). Greater green fluorescence and superior transfection efficiency of PAN_FAM_ in comparison to the FAM-siRNA/Lipofectamine in 4T1 cells was clearly evident within 6 h post-treatment. Additionally, the PAN_FAM_ caused morphological deformation of 4T1 cells (Fig. 9B) which might be attributed to the cationic nature of the PAN particles.

The transfection efficiency was further confirmed by uptake study of the formulations, Exo(PAN_FAM_) and Exo(PAN_FAM+DTX_) to closely evaluate the effect of each component of the formulation. Being labeled with CM-DiI (red fluorescence probe) EF formulations, Exo(PAN_FAM-siRNA_) and Exo(PAN_FAM+DTX_) exhibited co-localized yellow signal inside the cells after cellular uptake, which provided an additional proof that FAM-siRNA is encapsulated inside the PAN (Fig. 9C,D). Also, the green fluorescence if not co-localized with the red fluorescence of the exosomes would indicate the release of FAM-siRNA from both the formulations, Exo(PAN_FAM-siRNA_) and Exo(PAN_FAM+DTX_). Interestingly, Exo(PAN_FAM-siRNA_) and Exo(PAN_FAM+DTX_) treated cells showed lesser fluorescence intensity than the PAN_FAM_, possibly due to delayed release of the payload caused by layering of the PAN particles with the EF. Further, the EF layering of PAN_FAM_ proved beneficial in maintaining the cellular morphology as evident upon comparison of Fig. 9B vs. Fig. 9C. This is attributed to the protection of cells by EF in Exo(PAN_FAM_) from the cationic nature of the PAN_FAM._ Lastly, it was also observed that the cells treated with Exo(PAN_FAM_) showing yellow fluorescence in their cytoplasm were healthier and retained their morphology and nuclear integrity in comparison to the cells treated with Exo(PAN_FAM+DTX_) (Fig. 9C vs. Fig. 9D) owing to the anti-tumor effect of DTX present in the formulation. It indicated cytoskeletal deformation and multinucleation in the 4T1 cells (as indicated by red arrow) attributed to the presence of DTX as has already been reported in our previous work^35^. Based upon literature evidences, the final formulation is expected to follow clathrin and dynamin mediated pathways for internalization into the cells. MDA MB 231 derived exosomal membrane enclosed PLGA nanoparticles loaded with DiO, were reported to follow the dynamin mediated pathway in MDA MB 231 cells^36^.

##### In vitro cytotoxicity assay

**Fig. 10:**
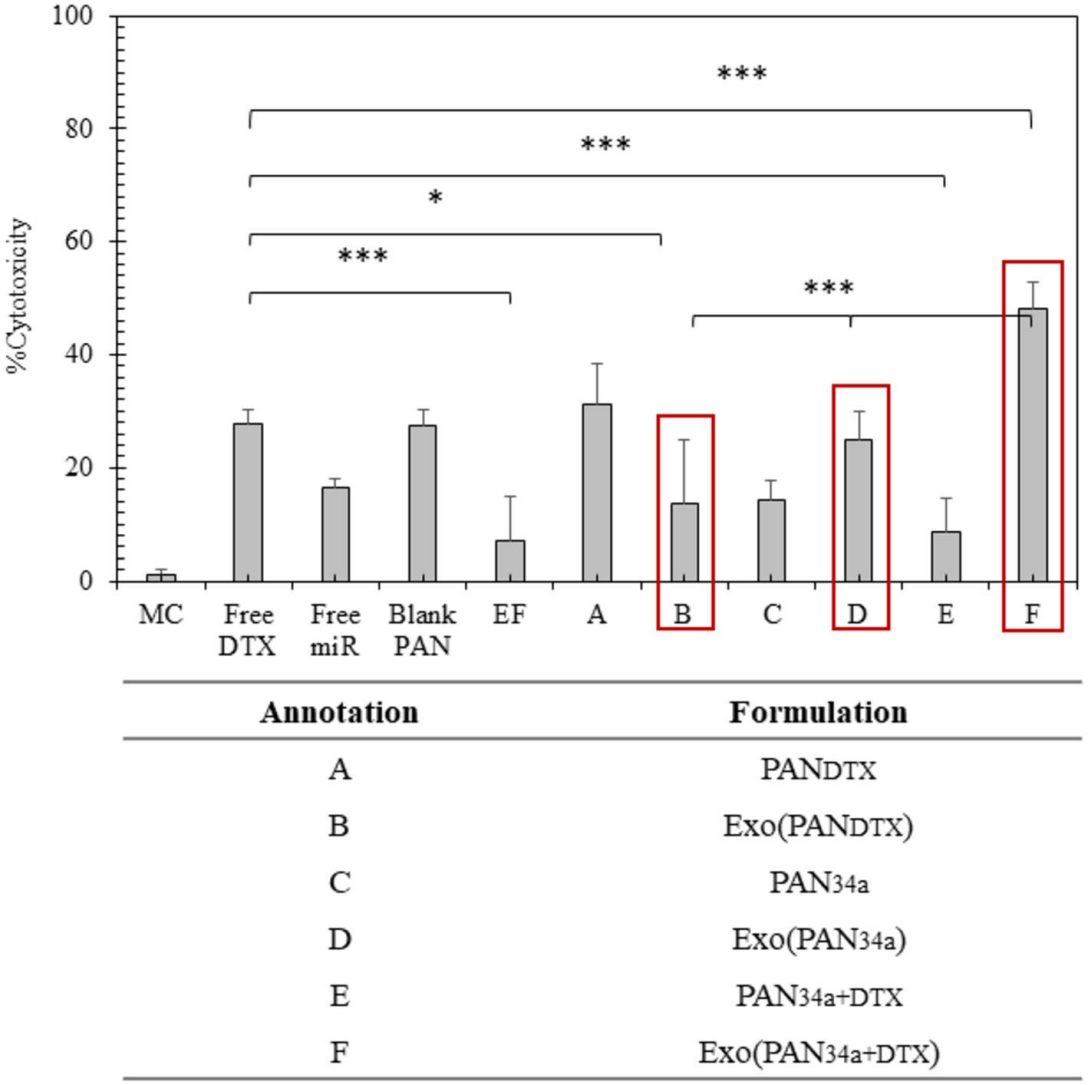
Anti-proliferative assay in 4T1 cells after 48 h. The cytotoxic effect of formulations A–F (as annotated) in comparison to the free DTX (40 ng/mL), free miR-34a (50 nM), free PAN, and EF (~ 12 μg) on 4T1 cells after being treated for 48 h. All data represented here is mean(n = 6) ± SD. One-way ANOVA with Tukey’s test was used for the determination of the statistical significance, where *p < 0.05, **p < 0.01 and ***p < 0.001.

It has already been reported by our group that cells treated for 48 h with DTX exhibited significant cytotoxicity based on the concentration of DTX and the IC_50_ of free DTX was found to be 480.04 ng/ml^35^. Figure 10, further confirmed that PAN_DTX_ (40 ng/ml, designated as A in the figure), PAN_34a_ (50 nM, designated as C in the figure) and their respective combination PAN_34a+DTX_ (designated as E in the figure) resulted in 31.20 ± 7.21%, 14.36 ± 3.48% and 8.74 ± 5.93% cytotoxicity respectively, whereas free PAN showed 27.51 ± 2.63% cytotoxicity.

It was also evident that complexation with miR-34a and miR-34a + DTX resulted in reduction of the positive charge of PAN which lowered the cytotoxicity of the formulations as compared to the PAN particles alone. The observed toxicity of PAN_DTX_ was further reduced to 13.76 ± 11.28% after getting layered with EF wherein, EF itself induced only 6.92 ± 8.08% cell death, which can be considered negligible. In comparison to Exo(PAN_DTX_), superior cytotoxicity was exhibited by Exo (PAN_34a_) and Exo(PAN_34a_ + DTX) with 25.06 ± 4.78% and 48.20 ± 4.59% after 48 h of the treatment.

##### Inflammatory cytokine release study

**Fig. 11:**
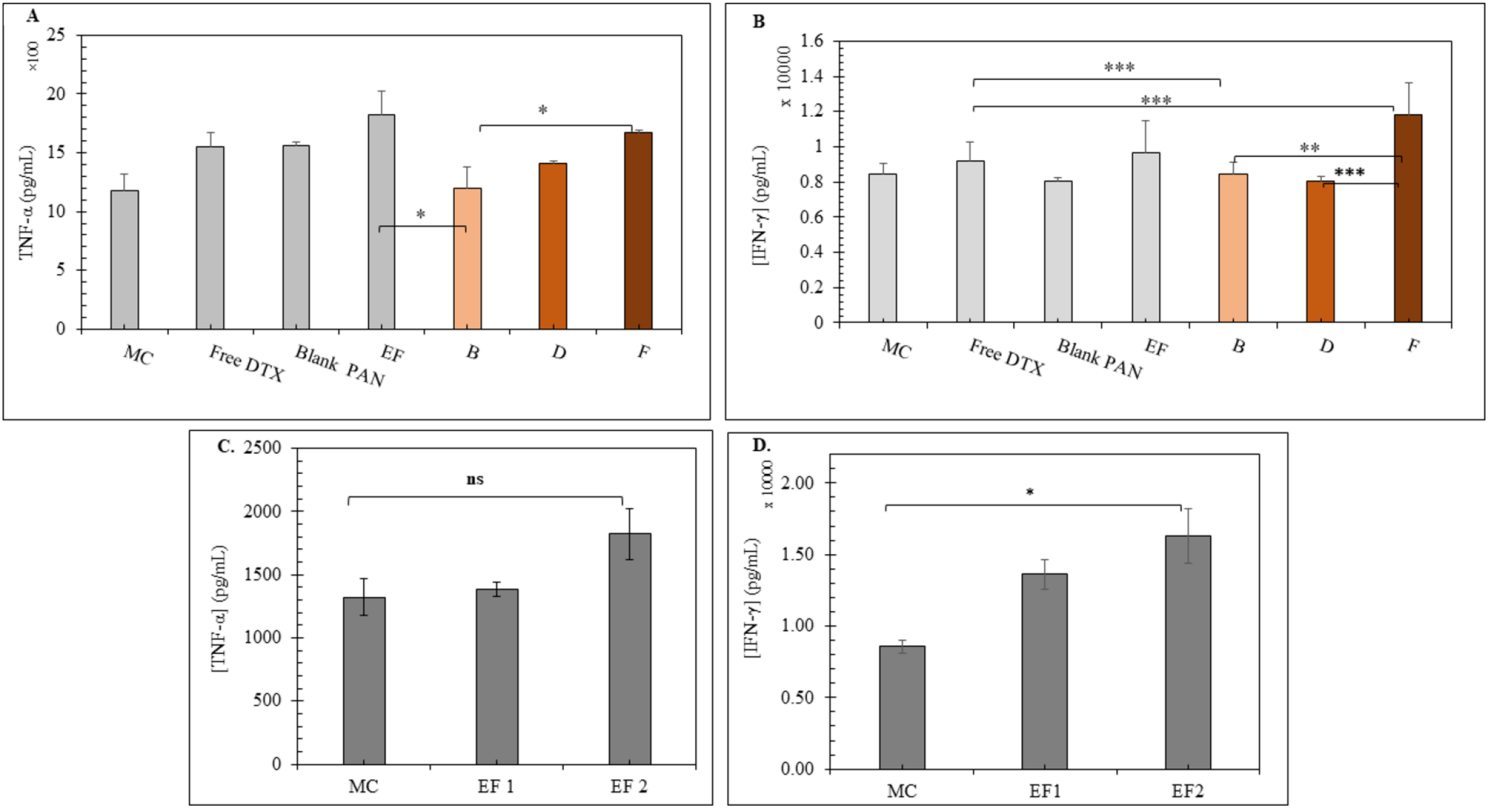
Effect of formulations (**B**), (**D**), and (**F**) and EF on the inflammatory soluble cytokine release by 4T1 cells, herein, formulation (**B**), (**D**), and (**F**) denote Exo(PAN_DTX_), Exo(PAN-miR34a) and Exo(PAN_34a+DTX_) respectively (**A**) TNF-α and (**B**) IFN-γ in the conditioned media of the cells treated with free DTX (40 ng/mL), blank PAN, EF, and formulations B, D, and F for 48 h. The release of (**C**) TNF-α and (**D**) IFN-γ by EF treatment in a concentration dependent manner in 4T1. Effect of EF1 (6 μg/mL) and EF2 (12 μg/mL) indicated the biological role of EF in 4T1 cells. All data represented here as (n = 6) ± SD, One-way ANOVA with Tukey’s test was used for the determination of the statistical significance, where *p < 0.05, **p < 0.01 and ***p < 0.001.

As indicated in Fig. 11A and B, 4T1 cells treated with free DTX showed elevated release of inflammatory cytokines, TNF-α and IFN-γ in comparison to the normal cells (without any treatment). Evidently, Blank PAN had non-significant impact on the 4T1 cells but blank EF exhibited immune-stimulant effect which was further highlighted in Fig. 11C,D. While comparing the effect of the different formulations on the cytokine release, Exo(PAN_34a+DTX_) showed prominent impact on 4T1 cells by releasing highest amount of TNF-α and IFN-γ with 1.20 ± 1.83 ng/mL and 11.8 ± 1.84 ng/mL respectively which was significantly higher in comparison to Exo(PAN_DTX_) and Exo(PAN_miR34a_). Interestingly, EF demonstrated immune-stimulant effect by increasing the release of both TNF-α and IFN-γ as indicated in Fig. 11C,D in a concentration dependent manner.

Although, elevated release of TNF-α is non-significant in Fig. 11C, elevation in IFN-γ was found significantly higher than the media control group in 4T1 cells. In comparison to EF1 (6 μg/mL), EF2 with 12 μg/mL equivalent protein resulted in 1.31 fold and 1.19 fold elevated TNF-α and IFN-γ release by the cells.

##### Gene expression analysis

**Fig. 12:**
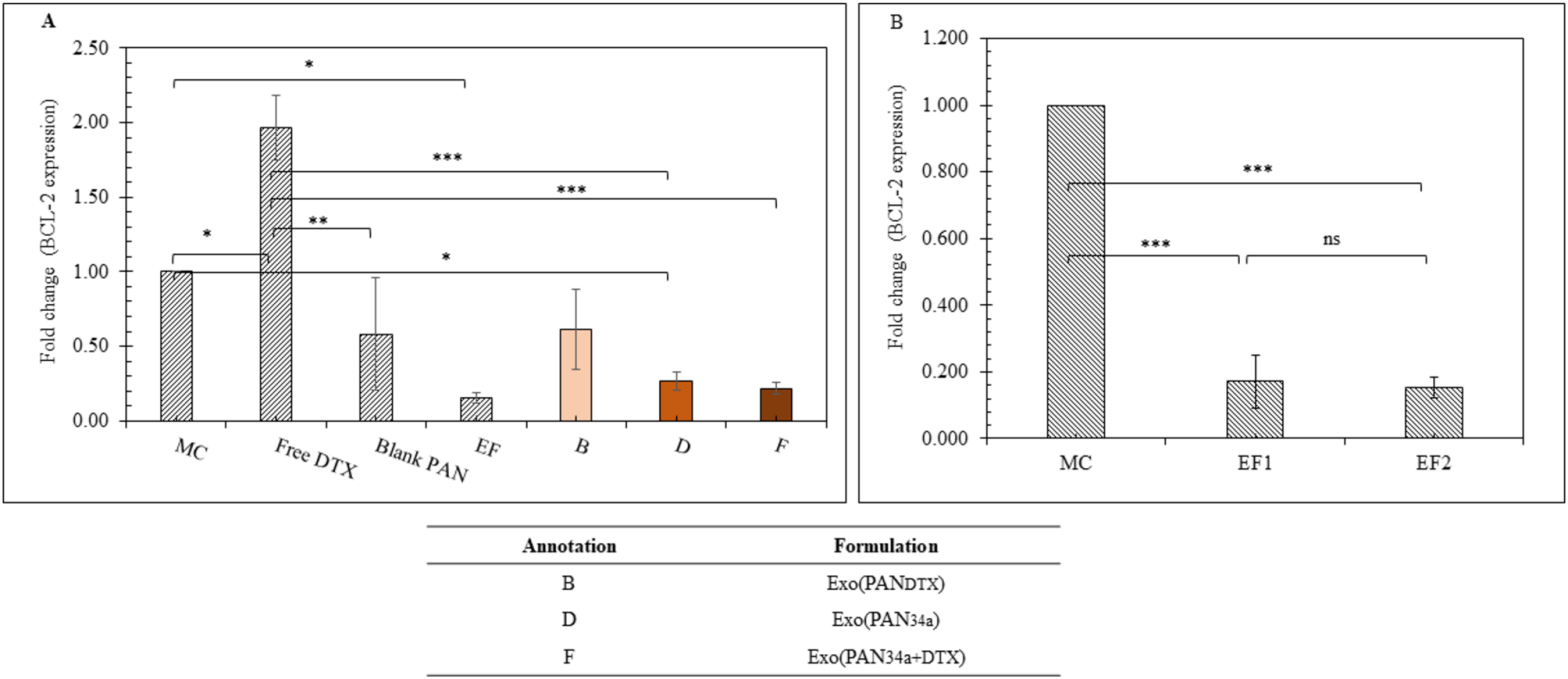
qRT-PCR analysis of 4T1 cells for the expression of miR-34a target gene BCL-2. (**A**) Effect of different formulations and, (**B**) effect of EF in a concentration dependent manner wherein, EF1 indicated ~ 6 μg/mL protein, and EF2 indicated ~ 12 μg/mL protein. All data are represented as mean (n = 3) ± SD and statistical comparison was performed by applying one-way ANOVA with Tukey multiple comparison test. *p < 0.05, **p < 0.01, and ***p < 0.001.

As indicated in Fig. 12, the overall effect of the formulations on the expression level of BCL-2 was ascertained. Figure 12A indicated that free DTX (40 ng/mL) elevated BCL-2 expression level in 4T1 cells, but the expression was decreased after treatment with blank PAN and EF by 1.7 and 6.5 fold respectively. Interestingly, Exo(PAN_DTX_) reduced the BCL-2 expression by 3.20 fold as compared to free DTX, but did not behave significantly different from that of the blank PAN. Whereas, Exo(PAN_34a_) and Exo(PAN_34a+DTX_) significantly reduced the BCL-2 expression in 4T1 cells in comparison to free DTX. As indicated by Fig. 12B, EF was also observed to suppress the BCL-2 expression significantly in 4T1 cells in a concentration independent manner.

## Discussion

The formation of PSA based core/shell nanoparticles involved assembly of the poly-cationic polymer, poly-allylamine hydrochloride, PAH by ionic crosslinking with multivalent anionic salts (e.g., HPO_4_^–2^ and H_2_PO_4_^–1^) into a metastable template to encapsulate the cargo (herein, DTX and miR-34a) to form the core, and then the shell material (exosomal fragment, EF) was deposited on the core by leveraging the electrostatic interaction between the reverse surface charges of the core and shell material^23^. After initial optimization of the buffer composition (Na_2_HPO_4_: KH_2_PO_4_ = 5:3 with [NaCl] = 10 mM), PAH concentration, and dilution, PAN particles prepared with a suitable R ratio (~ 1.21) provided desired size (122.25 ± 0.96 nm) and zeta-potential values (24 ± 4.35 mV) with characteristic loosely bound structure which is in alignment with the existing literature^29^. Recently, Doxorubicin (DOX) has been reported to self-associate in water upon increasing its concentration (0.1–100 μM) or concentration of polyanion like polystyrene sulphonate, PSS (0.48–48 mM) and formed complexes wherein, PSS acted as a template for concentrating DOX^37,38^. In our study, DTX in its supersaturated aqueous solution (log P 2.4, solubility ~ 12.4 μg/mL) carried slightly negative charge (− 3.5 ± 0.176 mV) at physiological pH which aided its encapsulation into the polycationic polymeric backbone of PAH resulting in 77.5% EE at pH 7.4. The efficient encapsulation of DTX in PAN was confirmed after dialysis of the formulation in water to allow the expulsion of the dissolved unentrapped DTX from the PAN_DTX_ particles (Fig. 3D). Further, the functional characterization by in-vitro release study of PAN_DTX_ supported the efficient encapsulation of DTX by showing a pH dependent release at pH 5.5 and at 7.4 (Fig. 8B). This characteristic release pattern observed at pH 5.5 is attributed to the fact that PAN dissembles at pH < 6 and pH > 9 to release the entrapped DTX since PAH (pKa 8.8) becomes deprotonated^29,39^. During the formulation development, it was evident that these PAN_DTX_ particles were stable at physiological pH (pH 7.2) since PAN_DTX_ was formed with reproducible %EE and maximum absolute intensity at pH 7.2, both of which decreased at pH 5.5 and almost diminished at pH 9.4 (Fig. 3B,C). Literature supports this observation wherein, Indocyanin green (ICG) with only one negative charge has been reported to be encapsulated in PAH-phosphate PSA with the help of hydrophobic interaction (~ 29%)^23,26^.

PAN has been well reported for efficient delivery of GFP siRNA in stably expressing GFP transfected A549 cells and provided endosomal (pH 6.5–4.5) release of GFP siRNA in the cells resulting in 60–65% inhibition of the GFP fluorescence in GFP-A549^29^. Based on the available reports, miR-34a was incorporated into PAN_DTX_ with slight modification in the protocol to utilize these PAN particles for co-loading the DTX and miRNA. For initial optimization, we utilized the cellular RNA as the cargo, wherein, we observed a constant surface potential of the PAN_RNA_ as the amount of RNA was varied (Fig. 4C,D). Increase in derived count rate along with decreased zeta-potential upon incorporation of RNA and DTX into the reaction mixture confirmed encapsulation of DTX and complexation of RNA to form PAN_(RNA+DTX)_ (Fig. 3E,F) Further, RNA was replaced with miR-34a and FAM-siRNA to form PAN_34a+DTX_ and PAN_FAM+DTX_ using the optimized method_._ While literature reports no significant change in particle size after complexation of siRNA with the PAN particles^28^), our result indicated an increase in particle size with 163.86 ± 12.89 nm and 164.85 ± 22.12 nm as compared to blank PAN (124.8 ± 30.61 nm) in PAN_34a+DTX_ and PAN_FAM+DTX_ respectively^29^. The efficient complexation of 0.5 μg miR-34a (~ 3.6 μM) in PAN_34a+DTX_ was evident as indicated in Fig. 5C at the optimized R ratio. This amount of miR-34a was used for all the further experiments for characterization and functional evaluation mentioned in this work. Also, the strong interaction between the miR-34a and PAH could be estimated by the observation made in the gel retardation assay in presence of the heparin (Fig. 5B). At R ~ 1.21 and ~ 0.2 μg miR-34 (1.45 μM), the complexation in PAN-miR 34a was found strong enough to resist the release the miRNA even after heparin treatment. Interestingly, PAN-miR 34a, prepared with compromised R ~ 121.4, resulted in inefficient complexation of miRNA (uncomplexed miRNA seen in lane 5; Fig. 5B), which further released most of the miRNA upon heparin treatment (lane 8 in the same gel; Fig. 5B). This clearly indicated the efficiency of the PAN particles forming the core of the system that was designed to co-deliver both small molecule drug, DTX and miRNA. Once the core of the formulation was optimized, the preparation and characterization of the shell was undertaken.

As the shell, the RAW 264.7 cell derived EF were prepared and characterized for particle size, zeta-potential and morphology. Exosomal extrusion and sonication methods for preparing EF are well reported in literature along with reports of an enhanced particle size of EF in one of the studies (351 ± 58.33 nm with high PDI 0.29 ± 0.01) which could be correlated well with our observation as well^19^. This is possibly the exosomal tendency to reseal and regain their structural integrity; which may result in the increase in particle size of the empty exosomes after extrusion. The single particle analysis by FESEM (n = 100) indicated heterogeneous size distribution of the EF with an average diameter of 180.73 ± 127.71 nm and DLS analysis of the same indicated 0.26 ± 0.06 PDI and − 11.3 mV zeta-potential. The prepared Exo(PAN_34a+DTX_), formulation demonstrated surface-potential equivalent to the EF, i.e., − 7.23 ± 2.75 mV, which is in line with the previous reports^19,36,40,41^. The change in the surface potential was in accordance with the reported literature but the particle size observed clearly differed from reported studies with the observed high particle size in FESEM and polydispersity by DLS. The final core/shell formulation, Exo(PAN_34a+DTX_) was significantly different from EF as well as PAN_34a+DTX_ as indicated by Fig. 7. The distinct change in morphology and compactness of formulation in comparison to blank PAN and EF as observed in FESEM is similar to that reported for ICG containing nanoparticle assembled capsule (NAC) further layered with the SiO_2_ nanoparticles^24^.

Once the final formulation was optimized, the Exo(PAN_34a+DTX_) was further evaluated for transfection efficiency and uptake in 4T1 cells. Both Exo(PAN_FAM+DTX_) and Exo (PAN_FAM_) were prepared and investigated in the studies. The co-localization study exhibited yellow fluorescence in the cells owing to the localization of CM-DiI (red) labeled EF and FAM-siRNA (green), confirming their being present in the same formulation. A similar co-localization study has been reported for the encapsulation of GFP-siRNA in the Rhodamine green labeled PAN particles^29^ and DiI (red) labeled exosome enclosed PLGA nanoparticles wherein, PLGA has been stained with DiO (green)^42^. Additionally, the observation was further supported by reduced green fluorescence and predominance of yellow fluorescence in Exo(PAN_FAM-siRNA_) and Exo(PAN_FAM+DTX_) treated cells than the PAN_FAM_, possibly due co-localization caused by the layering of the PAN_FAM_ particles with the EF (Fig. 9). Also, the detailed optimization of the process parameters has helped to assess the benefits of using the EF to reduce the cytotoxic effect of PAN_FAM_ which ensures layering of the particles with the EF. In fact a recent study indicated that nanoformulations containing the cationic excipients needs to consider the detailed optimization of the process parameters in order to eliminate the drawbacks of the cationic lipids or polymer in the final formulations^21,22^.

The efficiency of the formulations was further evaluated by in vitro anti-proliferative assay and anti-inflammatory cytokine release. Exo(PAN_34a+DTX_) containing 40 ng/mL DTX and 50 nM miR-34a was able to produce ~ 2.4 folds’ greater cytotoxicity in 4T1 cells (Fig. 10). Exo(PAN_34a+DTX_) also proved to enhance the inflammation in cells as indicated by the released TNF-α and IFN-γ in the media in comparison to the untreated cells in media, although the difference was not significant in case of TNF-α. TNF-α is an inflammatory cytokine and is expected to be increased by immune stimulation, but the functional duality of TNF-α only could be confirmed by the receptors present in the vicinity^43–45^. In our previous study, we reported that RAW 264.7 cell derived exosomes suppressed β1-integrin expression and increased the Cleaved Caspase 3/Caspase 3 ratio in TNBC 4T1 cells in a dose dependent manner^20^. As EF are prepared from the RAW 264.7 derived exosomes, these also exhibit similar effect on the 4T1 cells. It is well reported that TNF-α and IFN-γ are proinflammatory cytokines that upregulate the NF-κB mediated apoptotic pathway along with suppression of BCL-2 expression^46^. It is presumptive that EF, being obtained from macrophage derived exosomes also induce apoptosis in 4T1 cells by stimulating the expression of TNF-α and IFN-γ. DTX has been reported to initially enhance the TNF-α release from breast cancer cells in a time-dependent manner but reduces the TNF-α release in a concentration dependent manner within 48h^47^. On the contrary, IFN-γ release was profoundly significant in case of Exo(PAN_34a+DTX_) in comparison to the Exo(PAN_DTX_), Exo (PAN-miR 34a) and free DTX which indicated the efficiency of the formulation towards immune stimulation which could trigger the T-cell responses (TCR) to mediate the cancer cell cytotoxicity. The possibility could be hypothesized but can be only confirmed with more detailed studies. The final formulation evidently was able to produce anti-cancer effect and the novel approach utilized here successfully overcame the limitation of cytotoxicity triggered by the cationic nature of the PAN formulations by layering them with EF owing to their distinct immune-stimulant nature and high uptake efficiency. Comparing Figs. 10 and 11A, it can be concluded that the Exo(PAN_34a+DTX_) showed superior cytotoxic and immune-stimulant effect than the Exo(PAN_DTX_) and Exo (PAN-miR 34a) formulations. Additionally, the observed suppression of BCL-2 expression by Exo(PAN-miR34a) and Exo(PAN_34a+DTX_) could be correlated with the individual effect of the DTX and miR34a, as reported in the literature^4,48,49^. While, DTX has shown to regulate BAX/BCL-2 ratio instead only suppressing the BCL-2 expression in a dose and time-dependent manner, miR-34a is well reported to downregulate the BCL-2 translation in cancer as well as neurodegenerative diseases^12,50–52^. In fact, a cationic BSA (CBSA) coated nanocarrier of miR-34a and DTX has already been reported to deliver the payload in cytosol in a caveolae mediated pathway which was able to suppress the BCL-2 expression in both in vitro and in vivo TNBC model^53^. miR-34a mediated BCL-2 suppression aided in the chemo-sensitivity of DTX, which was also observed in our case. As indicated in Fig. 12A, the BCL-2 expression in Exo(PAN-miR34a) and Exo(PAN_34a+DTX_) showed significant downregulation in comparison to both Exo(PAN_DTX_) and free DTX. In addition to their role in release of TNF-α and IFN-γ (Fig. 11B,C), the prominent effect of the EF in BCL-2 downregulation (Fig. 12B) is also evident, but the detailed mechanism is yet to be investigated. Hence, exosomal membrane enclosed PAN particles could be utilized to co-deliver both the small molecule and macromolecule.

## Conclusion

In this study, functionally active RAW EF were explored to develop exosomal membrane enclosed miR-34a and DTX co-loaded PAN particles, Exo(PAN_34a+DTX_). The novelty in this strategy was utilizing the anionic exosomal fragment to enclose the cationic polyamine salt aggregate of DTX and miR-34a (PAN_34a+DTX_) to neutralize the residual cationic charge on the PAN particles. The use of exosomal fragment proved beneficial in enhancing the cellular uptake and its inherent apoptotic response to TNBC 4T1 cells. The study indicated superior in vitro efficacy of the Exo(PAN_34a+DTX_) which might be attributed to incorporation of miR-34a and the ability of the PAN particles to release the payload by proton-sponge effect in a pH dependent manner. In fact, the Exo(PAN_34a+DTX_) formulation exhibited greater cytotoxicity in 4T1 cells in comparison to Exo(PAN_DTX_) that is, 48.20 ± 4.59% vs. 13.76 ± 11.28%, indicating synergistic effect of miR-34a and DTX in 4T1 cells. This new formulation strategy of using exosomal layering of the co-loaded polymeric nano-formulation revealed beneficial effect of the RAW EF with its characteristic anti-proliferative activity along with an easy uptake of the PAN_34a + DTX_ wherein, PAN released its payload in a pH dependent manner.

## Supplementary Information

Supplementary Information.

## Data Availability

Data would be provided on genuine request. The corresponding author of the paper could be contacted for the same.
