# Supplementary material for: Exosomal fragment enclosed polyamine-salt nano-complex for co-delivery of docetaxel and mir-34a exhibits higher cytotoxicity and apoptosis in breast cancer cells
Source: Sci Rep. 2024 Sep 17;14:21669. doi: 10.1038/s41598-024-72226-0 (PMC11408524; doi:10.1038/s41598-024-72226-0)
Supplement: Supplementary file 1 — Supplementary Information. [file 41598_2024_72226_MOESM1_ESM.docx]

***Supplementary Data***

**Exosomal fragment enclosed Polyamine-salt nano-complex for co-delivery of Docetaxel and miR-34a exhibits higher cytotoxicity and apoptosis in breast cancer cells**

Moumita Basak, Mrunal Kulkarni, Saibhargav Narisepalli, Deepak Chitkara, Anupama Mittal^*^

Department of Pharmacy, Birla Institute of Technology and Science (BITS PILANI), Pilani, Rajasthan, INDIA-333031

*All correspondence relating to this paper should be addressed to:

**Corresponding author**

Dr. Anupama Mittal,

Department of Pharmacy,

Birla Institute of Technology and Science (BITS), Pilani, Pilani Campus

Pilani, Rajasthan, INDIA-333 031

Phone: +91 1596 515708

Mobile number: +91 9660876009

Email ID: [anupama.mittal@pilani.bits-pilani.ac.in](mailto:anupama.mittal@pilani.bits-pilani.ac.in)

ORCID ID: [0000-0003-3344-9579](http://orcid.org/0000-0003-3344-9579)

**BLOT 1**

**BLOT 2**

**Figure S1:** Representative stain-free, developed and merged blots for ALIX, TSG101 and β-actin. The portion of the blots highlighted in yellow colored box is relevant for this study. The remaining part of each blot has already been published in our previous work (1). Herein, CL_F_, EL, EF, PL, and NC indicate RAW 264.7 cell lysate, exosome lysate, exosome fragments, protein ladder and negative control.

References

1. Basak, M., Narisepalli, S., Salunkhe, S. A., Tiwari, S., Chitkara, D., and Mittal, A. (2024) Macrophage derived Exosomal Docetaxel (Exo-DTX) for pro-metastasis suppression: QbD driven formulation development, validation, in-vitro and pharmacokinetic investigation. *Eur. J. Pharm. Biopharm.* **195**, 114175
